# The GBA1 K198E Variant Is Associated with Suppression of Glucocerebrosidase Activity, Autophagy Impairment, Oxidative Stress, Mitochondrial Damage, and Apoptosis in Skin Fibroblasts

**DOI:** 10.3390/ijms25179220

**Published:** 2024-08-25

**Authors:** Laura Patricia Perez-Abshana, Miguel Mendivil-Perez, Marlene Jimenez-Del-Rio, Carlos Velez-Pardo

**Affiliations:** 1 Neuroscience Research Group, University of Antioquia, University Research Headquarters, Calle 62#52-59, Building 1, Laboratory 411/412, Medellin 050010, Colombia; lpatricia.perez@udea.edu.co (L.P.P.-A.); miguel.mendivil@udea.edu.co (M.M.-P.); marlene.jimenez@udea.edu.co (M.J.-D.-R.); 2 Faculty of Medicine, University of Antioquia, University Research Headquarters, Calle 62#52-59, Building 1, Laboratory 411/412, Medellin 050010, Colombia; 3 Faculty of Nursing, University of Antioquia, University Research Headquarters, Calle 62#52-59, Building 1, Laboratory 411/412, Medellin 050010, Colombia; 4 Institute of Medical Research, University of Antioquia, University Research Headquarters, Calle 62#52-59, Building 1, Laboratory 411/412, Medellin 050010, Colombia

**Keywords:** alpha-synuclein, apoptosis, autophagy, glucocerebrosidase, skin fibroblast, K198E variant, LRRK2, Parkinson

## Abstract

Parkinson’s disease (PD) is a multifactorial, chronic, and progressive neurodegenerative disorder inducing movement alterations as a result of the loss of dopaminergic (DAergic) neurons of the pars compacta in the substantia nigra and protein aggregates of alpha synuclein (α-Syn). Although its etiopathology agent has not yet been clearly established, environmental and genetic factors have been suggested as the major contributors to the disease. Mutations in the glucosidase beta acid 1 (*GBA1*) gene, which encodes the lysosomal glucosylceramidase (GCase) enzyme, are one of the major genetic risks for PD. We found that the GBA1 K198E fibroblasts but not WT fibroblasts showed reduced catalytic activity of heterozygous mutant GCase by −70% but its expression levels increased by 3.68-fold; increased the acidification of autophagy vacuoles (e.g., autophagosomes, lysosomes, and autolysosomes) by +1600%; augmented the expression of autophagosome protein Beclin-1 (+133%) and LC3-II (+750%), and lysosomal–autophagosome fusion protein LAMP-2 (+107%); increased the accumulation of lysosomes (+400%); decreased the mitochondrial membrane potential (∆Ψm) by −19% but the expression of Parkin protein remained unperturbed; increased the oxidized DJ-1Cys106-SOH by +900%, as evidence of oxidative stress; increased phosphorylated LRRK2 at Ser935 (+1050%) along with phosphorylated α-synuclein (α-Syn) at pathological residue Ser129 (+1200%); increased the executer apoptotic protein caspase 3 (cleaved caspase 3) by +733%. Although exposure of WT fibroblasts to environmental neutoxin rotenone (ROT, 1 μM) exacerbated the autophagy–lysosomal system, oxidative stress, and apoptosis markers, ROT moderately increased those markers in GBA1 K198E fibroblasts. We concluded that the K198E mutation endogenously primes skin fibroblasts toward autophagy dysfunction, OS, and apoptosis. Our findings suggest that the GBA1 K198E fibroblasts are biochemically and molecularly equivalent to the response of WT GBA1 fibroblasts exposed to ROT.

## 1. Introduction

Parkinson’s disease (PD) was initially described as a peculiar entity in six individuals by James Parkinson in 1817 [1]. Today, it has reached pandemic proportions [2,3] affecting over 8.5 million individuals worldwide (https://www.who.int, accessed on 8 July 2024 [4]). This disorder is a chronic and progressive neurodegenerative disorder mainly characterized by the following four cardinal motor alterations: rest tremor, bradykinesia, rigidity, and loss of postural reflexes [5] caused by the selective deterioration of dopaminergic (DAergic) neurons from the pars compacta of the substantia nigra [6] and the presence of Lewy’s intraneuronal inclusion bodies [7,8] mainly constituted by protein aggregates of alpha synuclein (α-Syn) [9]. Although the exact etiology is not yet known, environmental agents (e.g., pesticides (rotenone, ROT)) [10,11,12], gene mutations [13], or an interplay between both have been associated with PD [14]. However, the definitive etiopathogenic origin of PD is still unknown. Interestingly, genetic alterations in several genes such as glucosidase beta acid (*GBA1*), leucine-rich repeated kinase 2 (*LRRK2*), and synuclein alpha (*SNCA*), among others [15], have been implicated in functional neuronal alterations, such as mitochondrial oxidative phosphorylation, autophagy–lysosomal metabolism, ubiquitin–proteasome protein degradation, endoplasmic reticulum stress/unfolded protein response, and oxidative stress (OS), as major players in the molecular development of PD [16]. Therefore, delving into the molecular mechanism of DAergic neuron deterioration might provide a better understanding of PD [17]. Moreover, an understanding of the genetic factors influencing PD pathogenesis can have major impacts on therapeutic design and clinical care [18]. To this end, several in vivo and in vitro models have been used [19,20,21]. However, it has become possible to recapitulate the molecular complexities underlying the PD pathology by using skin fibroblasts, one of the most reliable and cost-effective models of this neurological disorder [22,23]. 

The glucosidase beta acid 1 (*GBA1*) gene contains 11 exons and 10 introns, spanning a 7.6 kb sequence on chromosome 1q21 encoding for the β-glucocerebrosidase (GCase) enzyme [24]. The GCase is a 497-amino-acid (a.a.) functional lysosomal hydrolase that metabolizes glucosylceramide (GlcCer) into glucose and ceramide or glucosyl sphingosine (GlcSph) into glucose and sphingosine (KEGG Enzyme EC 3.2.1.45). The three-dimensional structure of GCase comprises the following three non-contiguous folding domains: domain I (residues 1–27 and 383–414), a three-strand antiparallel β-sheet bordered by a loop and a perpendicular strand; domain II (residues 30–75 and 431–497) that contains two closely associated β-sheets that form an independent domain, which resembles an immunoglobulin (Ig) fold; domain III (residues 76–381 and 416–430), a (β/α)_8_ triosephosphate isomerase (TIM) barrel that works as the catalytic site of the enzyme [25]; the enzyme is synthesized in the rough endoplasmic reticulum (ER) and traverses the Golgi apparatus via a phosphatidylinositol-4-kinase (PI4K)-dependent pathway to the lysosome. Transit is mediated by a specific transporter, LIMP-2 (lysosomal integral membrane protein 2) [26,27]. Importantly, the GCase activity is dependent on the substrate-presenting co-factor saposin C, an essential 84-residue activator peptide [28]. Although no protein function has been attributed to the first two domains, docking studies have suggested that they are critically involved in GCaseSapoC interactions [29]. To date, 716 disease-causing GBA1 mutations have been described, including 535 missense/non-sense, 42 splicing, and 5 regulatory mutations, 55 and 20 small deletions and small insertions, respectively, 9 indels, as well as 15 and 3 gross deletions and gross insertions, respectively, and 32 complex mutations (The Human Gene Mutation Database, http://www.hgmd.org, accessed on 8 July 2024). Bi-allelic mutations result in negligible GCase enzyme activity and the autosomal recessive lysosomal storage disorder Gaucher’s disease (GD), in which GlcCer accumulates in various organs and cells (particularly macrophages) [30]. Although *GBA1* mutations do not cause a Mendelian form of PD, several studies have shown that heterozygous or homozygous *GBA1* mutations increase the risk of PD 20–30 fold and are found in all three structural domains of GCase [31]. However, the pathogenic mechanisms of GBA1 mutation-associated PD are not yet fully understood. Interestingly, though GBA1 PD is clinically, pathologically, and pharmacologically virtually indistinguishable from idiopathic PD [32], the genotype–phenotype correlation remains incomplete, e.g., [33]. Indeed, divergent phenotypes in family members harboring identical *GBA1* mutations have implicated the role of disease modifiers [34]. Moreover, different GBA1 mutations (e.g., N370S, D409H, and L444P) might contribute to clinical variations in PD [35]. However, the molecular mechanisms by which GBA1 mutations increase PD risk and their pleotropic effects remain largely unknown. Therefore, GBA1 has become a paradigm to establish pathways in neurodegeneration [31]. 

Among the several missense mutations reported so far, we have observed a significantly higher proportion of GBA1 mutation carriers in PD patients compared to healthy controls, primarily due to the presence of a population-specific mutation, such as the p.K198E variant, a single base substitution of a lysine to a glutamic acid at codon 198 (c.709A>G, g.4385A>G, exon 6, p.Lys198Glu) in a Colombian cohort [36]. Indeed, homozygous p.K198E (hereafter K198E) appears to be associated with GD type 2 (infantile, neuronopathic) [37], whereas heterozygous K198E represents a highly risk factor for PD [36]. Unfortunately, there are no data available to establish the molecular mechanism by which the GBA1 K198E variant might contribute to PD etiopathogenesis. Although mounting evidence suggests that GBA1/GCase-induced impairments connect to global lysosomal and autophagic dysfunction and oxidative stress through interaction with LRRK2, DJ-1, and α-Syn [38,39,40], further mechanistic studies are still needed to better understand this association. 

The autophagy–lysosomal system (ALS) is a complex mechanism for degrading intracellular cytosolic and organelles, involving more than 30 proteins [41,42]. Upon induction of autophagy (also referred to as “macroautophagy”), an isolating membrane envelops a section of the cytoplasm, generating the distinctive double-membraned organelle known as the autophagosome. The autophagosome formation process involves an initiation step, in which the mammalian target of rapamycin (mTOR), a ubiquitous serine-threonine protein kinase [43], is inhibited, resulting in unc-51-like autophagy activating kinase 1 (UKL1)-autophagy-related gene 13 (Atg13) pathway activation; nucleation of autophagic vesicles by the Beclin-1/vacuolar protein sorting 34 (VPS34)/VPS15/Agt14 complex; and expansion of autophagosomal membranes regulated by 2 ubiquitin-like conjugation pathways, ending with both Atg15/Atg12/Atg16 and insertion of the microtubule-associated protein 1 (MAP1) light chain 3-II (hereafter referred to as LC3-II) into autophagosomal membranes [44,45]. After the autophagosome–lysosome fusion, which produces an autolysosome, lysosomal enzymes such as GCase break down the contents (e.g., GlcCer, GlcSph) in this last vacuolar structure. However, whether loss of enzyme activity, gain of function due to enzyme misfolding, or loss of enzyme activity due to enzyme misfolding underlies the pathogenicity of the GBA1 K198E mutation is still not fully established. 

The present investigation aims to molecularly characterize the GBA1 K198E variant concerning its pathological role by enzymatic deficiency, oxidative stress, autophagy, and/or apoptosis-a regulated cell death [46] on skin fibroblasts in a heterozygote *GBA1* variant PD patient [36]. To this end, biochemical assays and molecular biology techniques, including flow cytometry and fluorescent microscopy analysis were used to inquire about the GBA1 K198E variant effects on PD skin fibroblast. We found that the GBA1 K198E fibroblasts but not WT skin fibroblasts showed (i) reduced catalytic activity of mutant GCase (but its expression levels of GCase were not affected); (ii) an increase in the acidification of autophagy vacuoles (e.g., autophagosomes, lysosomes, and autolysosomes); (iii) an increase in the expression of autophagosome proteins such as Beclin-1 and LC3-II and lysosomal–autophagosome fusion protein LAMP-2; (iv) an increase in the accumulation of lysosomes; (v) a decrease in mitochondrial membrane potential (∆Ψm), but (vi) the expression of Parkin protein was unperturbed; (vii) an increase in oxidized DJ-1Cys106-SOH, as evidence of oxidative stress; (viii) an increase in phosphorylated LRRK2 at Ser935 along with phosphorylated α-synuclein (α-Syn) at pathological residue Ser129; (ix) an increase in the executer apoptotic protein caspase 3 (cleaved caspase 3, CC3). Although exposure of WT fibroblasts to environmental neutoxin rotenone (ROT, 1 μM) exacerbated the autophagy–lysosomal system, oxidative stress, and apoptosis markers, ROT moderately increased those markers in GBA1 K198E fibroblasts. We conclude that the K198E mutation endogenously primes skin fibroblasts toward autophagy dysfunction, OS, and apoptosis. Our findings suggest that the GBA1 K198E fibroblasts behave biochemical and molecularly equivalently to the response of WT GBA1 fibroblasts exposed to rotenone. 

## 2. Results

### 2.1. GBA1 K198E Variant Dramatically Inactivates the Catalytic Enzymatic Activity of GCase, but Did Not Affect the Protein Expression Levels

**Figure 1:**
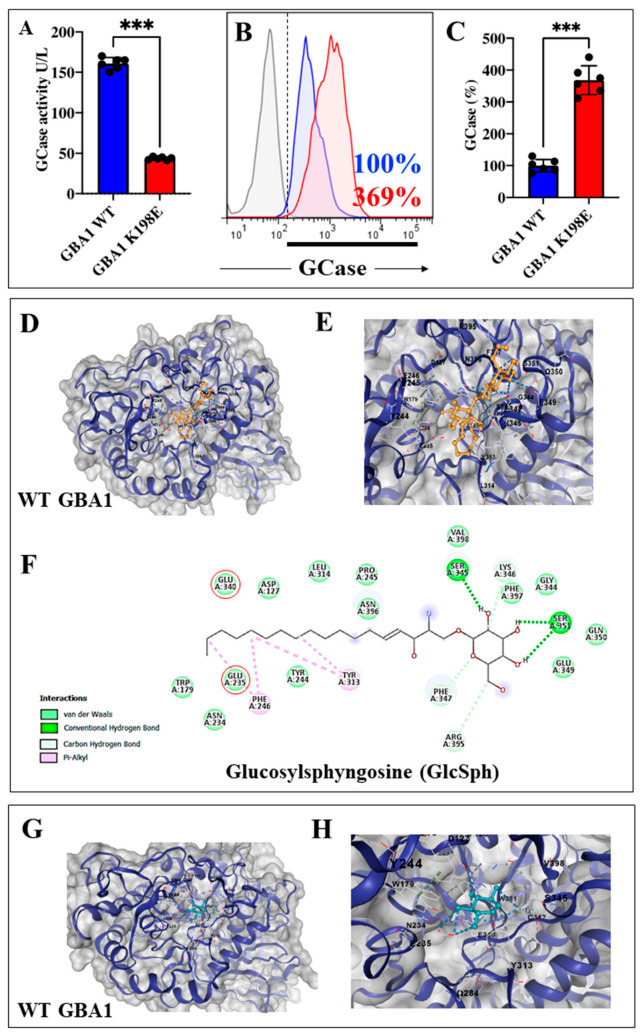
Enzyme activity and expression levels of glucocerebrosidase (GCase) in fibroblasts bearing the mutation GBA1 K198E with in silico molecular docking of glucosylsphingosine (GlcSph) and GCase. (**A**) Enzyme activity of GCase activity in WT GBA1 (blue bar) and GBA1 K198E fibroblasts (red bar). (**B**) Protein expression levels of glucocerebrosidase (GCase) in WT GBA1 (blue curve) and GBA1 K198E fibroblasts (red curve) assessed by flow cytometry. (**C**) Quantification of GCase expression levels. Numbers in histograms represent positive cellular population for the tested marker. (**D**) Representative CB-Dock2 3D images showing the molecular docking of WT GCase (created by Alphafold2) with GlcSph (PubChem CID: 5280570). (**E**) Representative enlarged image of (**D**) showing the molecular docking of WT GCase with GlcSph. (**F**) Two-dimensional diagram showing conventional hydrogen bond between GCaseGlcSph interaction. (**G**) Representative CB-Dock2 3D images showing the molecular docking of WT GCase with conduritol-B-epoxide (CBE, CID: 136345). (**H**) Representative enlarged image of (G) showing the molecular docking of WT GCase with CBE. The data are presented as mean ± SD of two independent experiments in triplicated (dots in bar). One-way ANOVA followed by Tukey’s test. Statistically significant differences *** *p* < 0.001.

Previous data have shown that *GBA1* mutations diminish GCase activity [47], either by reducing enzyme activity via alteration of catalytic properties, stability, change in substrate affinity, and modifier interactions [48] or by reducing the lysosomal enzyme concentration that results in proteasomal degradation of the protein due to increased protein folding [49]. Therefore, we initially wanted to determine the enzyme activity and protein expression levels of GCase in wild-type (WT) and GBA1 K198E fibroblasts. Figure 1A shows that the GCase enzyme activity in GBA1 K198E fibroblasts diminished by −70% when compared to GCase activity in WT fibroblasts. In contrast, mutant fibroblasts increased the GCase protein expression levels by +269% (3.68-fold increase) with respect to control fibroblasts (Figure 1B,C). We further calculated the theoretical binding affinity of the natural substrate glucosyl sphingosine (GlcSph, a deacylated analogue of GlcCer) to the catalytic pocket of GCase. In silico molecular docking analysis shows that GlcSph binds to GCase with high affinity (Vina Score (VS) −6.1 kcal/mol, Figure 1D,E), involving the critical residues Glu235 and Glu340 located at the active site of the enzyme (Figure 1F, Table 1) [50]. For validation purposes, we also calculated the covalent binding affinity of the well-known GCase inhibitor conduritol-B-epoxide (CBE), [51]. Effectively, CBE binds to GCase with high affinity energy (VS −5.7 kcal/mol, Figure 1G,H), thereby interacting with 54% identical amino acid (a.a., 12/22) as observed with GlcSph ( Table 1). Surprisingly, molecular docking analysis revealed that neither GlcSph nor CBE (used as a control) bind to the catalytic pocket of K198E GCase (Table 1). 

### 2.2. GBA1 K198E Shows an Acute GCase Deficiency in the Autophagy–Lysosome System

**Figure 2:**
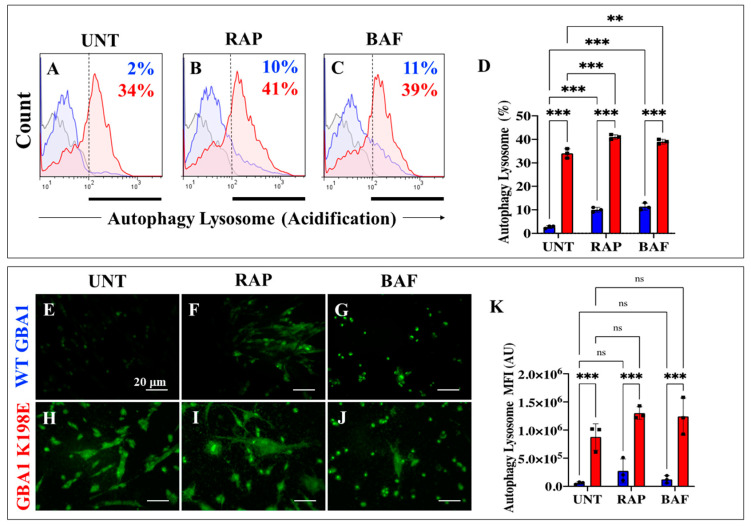
GBA1 K198E variant induces acute GCase deficiency in the autophagy–lysosome system reflected as acidification of autophagosomes, lysosomes, and autolysosomes in untreated or treated fibroblast with rapamycin (RAP) or bafilomycin A1 (BAF). (**A**) Representative flow cytometry histograms showing the autophagy–lysosome acidification in untreated WT (blue curve) and GBA1 K198E fibroblasts (red curve), (**B**) WT and GBA1 K198E fibroblasts treated with rapamycin (RAP, 10 nM) or (**C**) bafilomycin A1 (BAF, 10 nM). (**D**) Quantitative analysis of autophagy–lysosome (acidification)-positive cells. (**E**–**G**) Representative immunofluorescence images showing autophagy–lysosome acidification in (**E**) untreated WT fibroblasts, (**F**) treated with rapamycin (RAP, 10 nM) or (**G**) treated bafilomycin A1 (BAF, 10 nM). (**H**–**J**) Representative immunofluorescence images showing autophagy–lysosome acidification in (**H**) untreated GBA1 K198E fibroblasts, (**I**) treated with rapamycin (RAP, 10 nM) or (**J**) treated bafilomycin A1 (BAF, 10 nM). (**K**) Quantitative analysis of autophagy lysosome as mean fluorescence intensity (MFI). Numbers in histograms represent positive cellular population for the tested marker. The histograms and photomicrographs represent 1 out of 3 independent experiments (n = 3). The data are presented as mean ± SD of three independent experiments (dots in bar). One-way ANOVA followed by Tukey’s test. Statistically significant differences: ** *p* < 0.01, *** *p* < 0.001; ns = not significant. Image magnification: 200×.

Several studies have shown that GBA1 variants provoke GCase deficiency in the autophagy–lysosome system [52]. Therefore, we evaluated the effect of GBA1 K198E on the autophagy–lysosome process in WT and mutant fibroblasts. For comparative purposes, we included the autophagy inducer rapamycin (RAP) [53] and the inhibitor bafilomycin A1 (BAF) [54]. Figure 2 shows that WT GBA1 fibroblasts presented a low basal acidification in autophagosome, lysosome, and autolysosome organelles (Figure 2A,D,E,K), whereas RAP and BAF provoked an increase in organelle acidification in WT cells by +400% (Figure 2B–D,F,G,K). On the other hand, GBA1 K198E increases the acidification and accumulation of those organelles in mutant fibroblasts by +1600% (Figure 2A,D,H,K) compared to WT fibroblasts. However, RAP and BAF reagents slightly increased the acidification of the autophagy organelles in GBA1 K198E fibroblasts by +21% (Figure 2B,D,I,K) and +15% (Figure 2C,D,J,K), respectively, compared to untreated mutant fibroblasts. Of note, while the GBA1 K198E induced an increase in the acidification and accumulation of autophagy organelles (e.g., autophagosome, lysosome, and autolysosome, Figure 2H,K), treatment of mutant fibroblasts with RAP provoked the acidification and accumulation of autophagosomes and lysosomes (Figure 2I,K), and mutant fibroblasts treated with BAF induced acidification of autophagosomes only (Figure 2J,K). 

### 2.3. GBA1 K198E Increases the Expression of Beclin-1, LC3-II, and LAMP-2 in Fibroblasts

**Figure 3:**
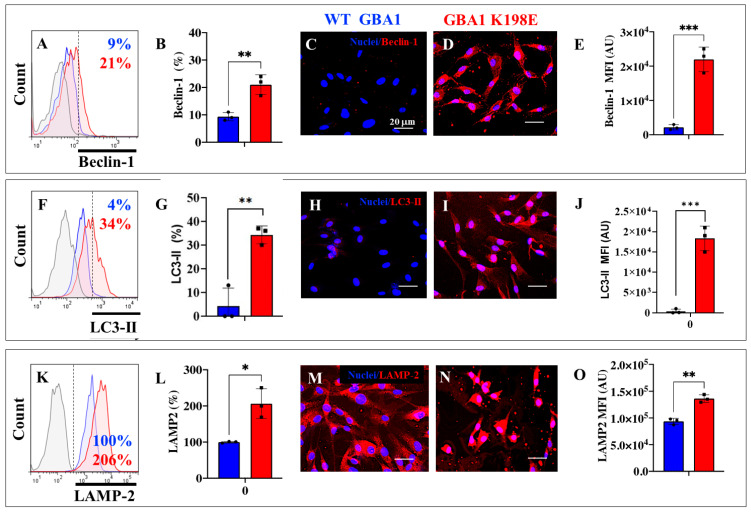
GBA1 K198E variant upregulates expression of autophagic Beclin-1, LC3-II, and LAMP-2 proteins in fibroblasts. (**A**) Representative flow cytometry histogram analysis showing Beclin-1 expression in WT-GBA1 (blue curve) and GBA1 K198E fibroblasts (red curve). (**B**) Quantitative (%) analysis of Beclin-1 expression in WT (**blue bar**) and GBA1 K198E fibroblast (red bar); (**C**) representative immunofluorescence image showing Beclin-1 reactivity in WT GBA1 and (**D**) K198E GBA1 fibroblasts (red fluorescence). (**E**) Quantitative (MFI) analysis of Beclin-1. (**F**) Representative flow cytometry histogram analysis showing LC3-II expression in WT GBA1 (blue curve) and GBA1 K198E fibroblasts (red curve). (**G**) Quantitative analysis of LC3-II in WT (blue bar) and GBA1 K198E fibroblasts (red bar). (**H**) Representative immunofluorescence images showing LCIII-2 reactivity in fibroblasts WT-GBA1 and (**I**) GBA1 K198E fibroblasts (red fluorescence). (**J**) Quantitative analysis of LC3-II. (**K**) Representative flow cytometry histogram analysis showing LAMP-2 expression in WT-GBA1 (blue curve) and GBA1 K198E fibroblasts (red curve). (**L**) Quantitative analysis of LAMP-2 in WT (blue bar) and GBA1 K198E fibroblasts (red bar). Nuclei were stained with Hoechst 33342 (blue fluorescence). (**M**) Representative immunofluorescence images showing LAMP-2 reactivity in fibroblasts WT GBA1 and (**N**) GBA1 K198E fibroblasts (red fluorescence). Nuclei were stained with Hoechst 33342 (blue fluorescence). (**O**) Quantitative analysis of LAMP-2. Numbers in histograms represent positive cellular population for the tested marker. Histograms and photomicrographs represent one out of three independent experiments (n = 3). The data are presented as mean ± SD of three independent experiments (dots in bar). One-way ANOVA followed by Tukey’s test. Statistically significant differences: * *p* < 0.05; ** *p* < 0.01; *** *p* < 0.001. Image magnification; 200×.

The above observations prompted us to further evaluate the effect of the GBA1 K198E variant in the autophagy–lysosome system [44]. Due to the complexity of the system [41], we selected the following three key proteins involved in the molecular cascade of autophagy: Beclin-1, a scaffold in the Phosphoinositide 3-Kinase (PI3K) Class III C1 and C2 nucleation complexes, is involved in the formation of phagophore vesicles [55]; the autophagosomal marker microtubule-associated protein light chain 3 (LC3-II), localized to pre-autophagosomes and autophagosomes, is degraded by lysosomal hydrolytic enzymes following the fusion of autophagosomes with lysosomes [56]; and lysosomal-associated membrane protein-2 (LAMP-2), a lysosomal membrane protein that regulates macroautophagy and chaperone-mediated autophagy (CMA), promotes the fusion of autophagic vacuoles with lysosomes [57]. As shown in Figure 3, while WT GBA1 fibroblasts express basal levels of Beclin-1 (Figure 3A,B), GBA1 K198E increased Beclin-1 expression by +133% (Figure 3A,B) compared to control fibroblasts. A similar trend in Beclin-1 expression in WT and mutant fibroblasts was observed by fluorescent microscopy (Figure 3C–E). Like Beclin-1, WT fibroblasts express basal levels of LC3-II (Figure 3F,G) and LAMP-2 (Figure 3K,L), whereas GBA1 K198E augmented the expression of LC3-II (Figure 3F,G) and LAMP-2 (Figure 3K,L) by +750% and +107%, respectively, compared to WT fibroblasts (Figure 3G,L). Comparable data were obtained by fluorescent microscopy analysis (Figure 3H–J,M–O). 

### 2.4. GBA1 K198E Variant Increases the Accumulation of Lysosomes and Decreases the Mitochondrial Membrane Potential (∆Ψm) in Mutant Fibroblasts, while Rotenone Increases the Damage

**Figure 4:**
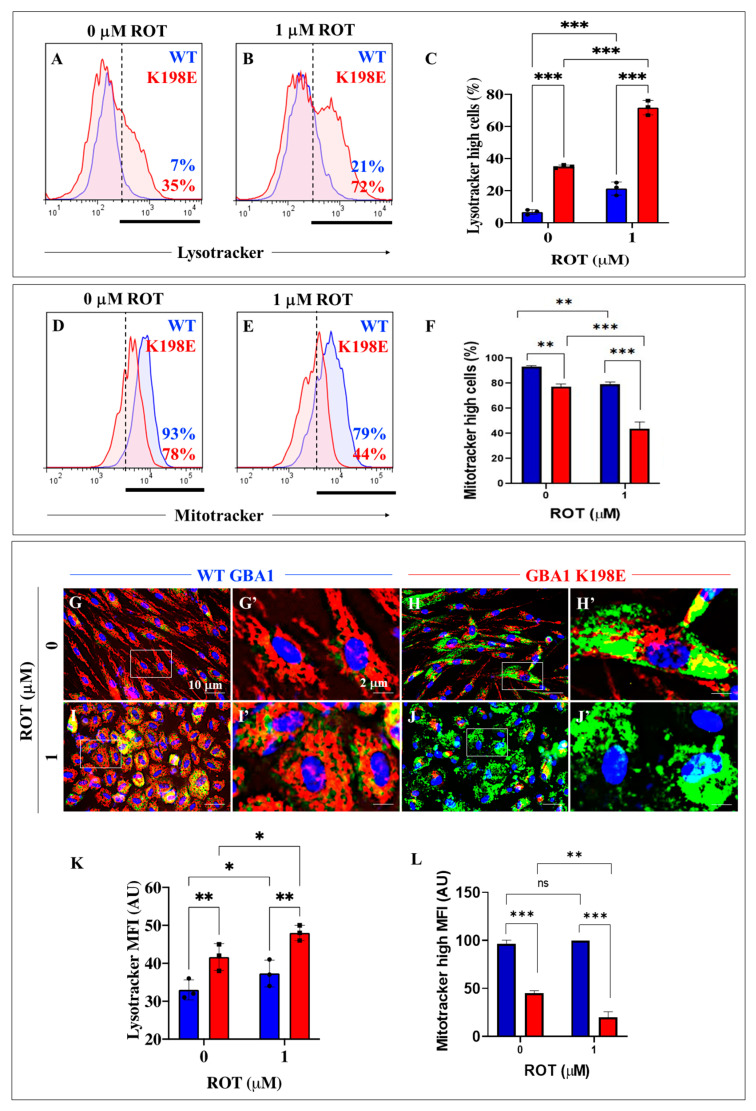
GBA1 K198E variant increases the accumulation of lysosomes and decreases the mitochondrial membrane potential (∆Ψm) in mutant fibroblasts, while rotenone aggravates the damage. (**A**) Representative flow cytometry histogram showing WT (blue curve) or GBA1 K198E fibroblasts (red curve) stained with Lysotracker^®^. (**B**) Representative flow cytometry histogram showing WT (blue curve) or GBA1 K198E fibroblasts (red curve) exposed to rotenone (ROT, 1 μM) and stained with Lysotracker^®^. (**C**) Percentage of Lysotracker^®^ stain-positive cells in untreated or treated WT (blue bar) and GBA1 K198E fibroblasts (red bar) with ROT. (**D**) Representative flow cytometry histogram showing WT (blue curve) or GBA1 K198E fibroblasts (red curve) stained with Mitotracker^®^. (**E**) Representative flow cytometry histogram showing WT (blue curve) or GBA1 K198E fibroblasts (red curve) exposed to rotenone (ROT, 1 μM) stained with Mitotracker^®^. (**F**) Percentage of Mitotracker^®^ stain-positive cells in untreated or treated WT (blue bar) and GBA1 K198E fibroblasts (red bar) with ROT. (**G**) Representative fluorescence microscopy image showing untreated WT fibroblasts stained with Lysotracker^®^ and Mitotracker^®^ (red fluorescence), (**G’**) close-up of image G (white line square); (**H**) representative fluorescence microscopy image showing untreated GBA1 K198E fibroblasts stained with Lysotracker^®^ (green fluorescence) and Mitotracker^®^ (red fluorescence). (**H’**) Close-up of image H (white line square); (**I**) representative fluorescence microscopy photograph showing WT fibroblasts treated with ROT (1 μM) and stained with Lysotracker^®^ (green fluorescence) and Mitotracker^®^ (red fluorescence), (**I’**) close-up of image I (white line square); (**J**) representative fluorescence microscopy photograph showing GBA1 K198E fibroblasts treated with ROT (1 μM) and stained with Lysotracker^®^ (green fluorescence) and Mitotracker^®^. (**J’**) Close-up of image J (white line square). (**K**) Quantification of the Lysotracker^®^ mean fluorescence intensity (MFI) in untreated or treated WT (blue bar) and GBA1 K198E fibroblasts (red bar) with ROT. (**L**) Quantification of the MitoTracker^®^ high mean fluorescence intensity (MFI) in untreated or treated WT (blue bar) and GBA1 K198E fibroblasts (red bar) with ROT. Nuclei were stained with Hoechst 33342 (blue fluorescence). Histograms and photomicrographs represent one out of three independent experiments (n = 3). The data are presented as mean ± SD of three independent experiments (dots in bar). One-way ANOVA followed by Tukey’s test. Statistically significant differences: * *p* < 0.05; ** *p* < 0.01; *** *p* < 0.001. Image magnification, 200×. ns = not significant.

Given that GCase not only localizes to lysosomes to maintain lysosomal membrane integrity [58] but also promotes the maintenance of mitochondrial complex I (CI) integrity and function [59], we wanted to investigate whether GBA1 K198E affected lysosomes and mitochondria in mutant fibroblasts. In addition, we wonder whether exposure to the environmental neurotoxin rotenone (ROT), a specific inhibitor of mitochondrial complex I [60,61], a GCase inhibitor and inducer of lysosome accumulation [39], could aggravate the GBA1 K198E-induced phenotype. To this end, WT and mutant fibroblasts were left untreated or treated with ROT (1 μM) and then analyzed with Lysotracker^®^ and Mitotracker^®^ reagents. Lysotracker Green^®^ is a cell-permeable, non-fixable, green fluorescent dye that stains acidic compartments within a cell, such as the lysosome, whereas Mitotracker^®^ Red is a cell-permeant probe that stains active mitochondrion in live cells. Lysotracker analysis shows that GBA1 K198E fibroblasts displayed an important endogenous increase in the percentage of accumulation of lysosomes (+400%, Figure 4A,C) compared to WT fibroblasts (Figure 4C). Upon ROT exposure, the percentage of lysosome detection increased by +200% in WT fibroblasts (3-fold increase, Figure 4B,C) and in mutant fibroblasts by +106% (Figure 4B,C) compared to untreated WT and mutant fibroblasts, respectively (Figure 4C). On the other hand, mitotracker analysis reveals that the GBA1 K198E fibroblast showed an important endogenous decrease in ∆Ψm (−19%, Figure 4D,F) compared to WT fibroblasts (Figure 4F). ROT induced a loss of ∆Ψm in WT fibroblasts by −15% (Figure 4E,4F) compared to untreated control fibroblasts (Figure 4F), whereas it provoked a loss of ∆Ψm in GBA1 K198E fibroblasts by −43% (Figure 4E,F) compared to untreated mutant fibroblasts (Figure 4F). Similar observations were obtained by fluorescence microscopy (Figure 4G–L).

### 2.5. GBA1 K198E Fibroblasts Show Parkin Protein Colocalization with Mitochodrial TOM20 Proteins

**Figure 5:**
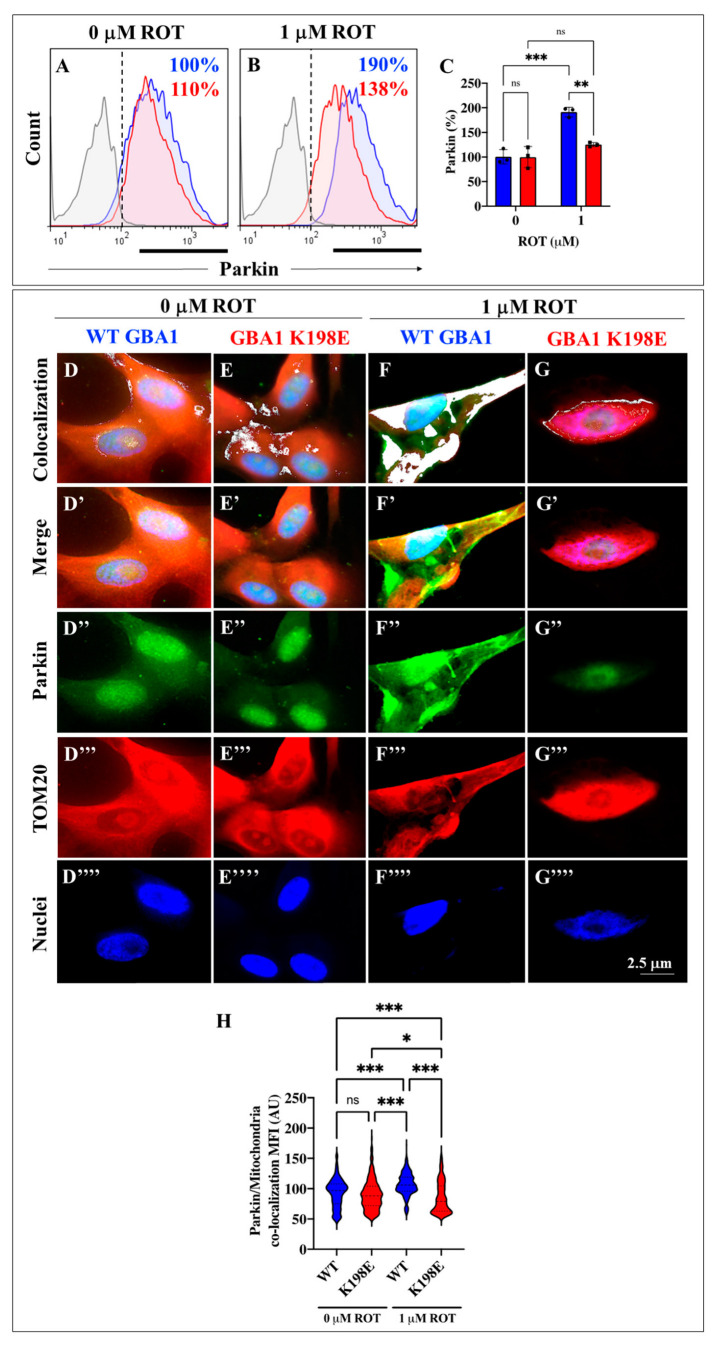
GBA1 K198E fibroblasts show unchanged expression levels of Parkin and mitochondrial colocalization of Parkin and TOM20 proteins, but Parkin shows a tendency to increase and mitochondrial colocalization upon rotenone exposure. (**A**) Representative flow cytometry histogram analysis showing the expression of Parkin protein in WT (blue curve) and GBA1 K198E fibroblasts (red curve). (**B**) Representative flow cytometry histogram analysis showing the expression of Parkin protein in WT (blue) and GBA1 K198E fibroblasts (red) upon rotenone (ROT, 1 μM) exposure. (**C**) Quantitative analysis of parkin expression. (**D**–**G**) Representative fluorescence merge density images of colocalization of Parkin and the translocase of the outer membrane of mitochondria 20 (TOM20) proteins in (**D**) WT fibroblasts (white fluorescence), (**E**) GBA1 K198E fibroblasts, (**F**) WT fibroblasts treated with rotenone (ROT, 1 μM), and (**G**) GBA1 K198E fibroblasts exposed to ROT (1 μM). (**D’**–**G’**) Representative fluorescence merge images in layer colocalization of Parkin (**D’’**–**G’**’, green fluorescence) with TOM20 proteins (**D’’’**–**G”’**, red fluorescence). Nuclei were stained with Hoechst 33342 (blue, **F’’’’**–**G’”’**). (**H**) Quantification of the Parkin/mitochondria mean fluorescence intensity (MFI). Flow cytometry histograms represent one out of three conducted experiments. The results are reported as the mean ± standard deviation of 3 independent experiments (dots in bar). Fluorescence microphotographs represent one out of three experiments (n=3). A one-way ANOVA, followed by Tukey’s test, was conducted for statistical analysis. Statistically significant variations are indicated by * *p* < 0.05, ** *p* < 0.01, *** *p* < 0.001; ns = no significance.

Previous studies have shown that Parkin protein, an E3 ubiquitin ligase, controls the lysosomal degradation of depolarized mitochondria through the mitophagy process [62]. We therefore investigated whether GBA1 K198E regulated the expression and/or localization of Parkin in fibroblasts. Figure 5 shows that WT and GBA1 K198E fibroblasts expressed Parkin at similar basal levels (Figure 5A,B). Under ROT exposure (at 1 μM concentration), WT and GBA1 K198E fibroblasts differentially expressed Parkin. While WT fibroblasts treated with ROT increased the expression of Parkin by +90% (Figure 5B,C), the GBA1 K198E fibroblasts exposed to ROT expressed Parkin to similar levels as untreated mutant fibroblasts (Figure 5A–C). Of note, GBA1 K198E fibroblasts treated with ROT reduced the expression of Parkin by −38% compared to WT exposed to ROT (Figure 5B,C). To track Parkin’s cellular position, we used the translocase of the outer membrane 20 (TOM20), a complex of proteins found in the outer mitochondrial membrane of the mitochondria [63]. Fluorescence microscopy analysis reveals that Parkin and TOM20 colocalized and distributed throughout the cytoplasm in WT (Figure 5D) but Parkin and TOM20 colocalized in “patchy” areas in GBA1 K198E fibroblasts (Figure 5E) that were not statistically different from WT fibroblasts (Figure 5H). When treated with ROT, Parkin and TOM20 highly colocalized in WT fibroblasts (Figure 5F,H), whereas the amount of colocalized proteins was almost inexistent in GBA1 K198E fibroblasts (Figure 5G,H). 

### 2.6. GBA1 K198E Fibroblasts Show High Oxidized DJ-1-Cys106-SOH into DJ-1 Cys106SO_3_

**Figure 6:**
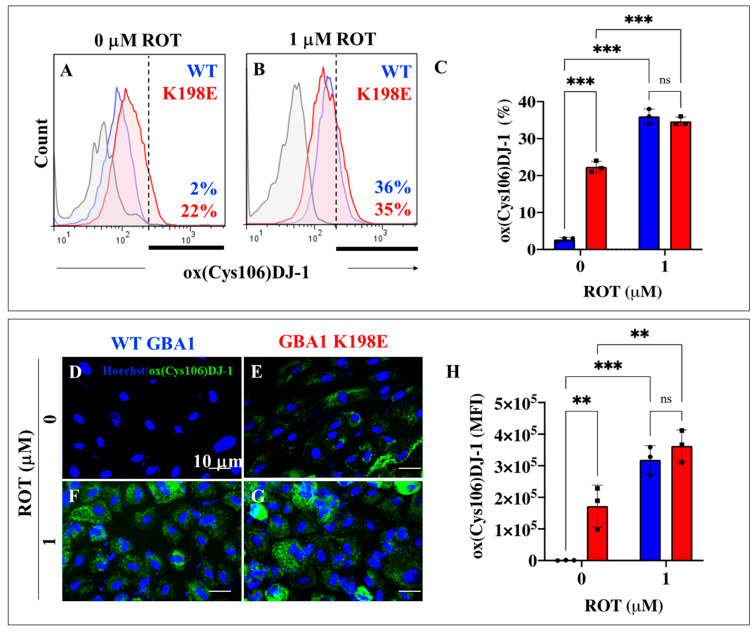
GBA1 K198E fibroblasts show an endogenously high percentage of oxidized DJ-1-Cys106-SOH into DJ-1 Cys106SO_3_. (**A**) Representative flow cytometry histogram analysis showing oxidized DJ-1 (Cys106-SO_3_) protein in WT (blue curve) and GBA1 K198E fibroblasts (red curve). (**B**) Representative flow cytometry histogram analysis showing oxidized DJ-1 (Cys106-SO_3_) protein in WT (blue color) and GBA1 K198E fibroblasts (red color) upon rotenone (ROT, 1 μM) exposure. Flow cytometry histograms represent one out of three conducted experiments. The results are reported as the mean ± standard deviation of 3 independent experiments. (**C**) Quantitative analysis of oxidized DJ-1 (Cys106-SO_3_) protein. (**D**) Representative immunofluorescence image showing oxidized DJ-1 protein (Cys106-SO_3,_ green fluorescence) in WT GBA1 fibroblasts. (**E**) Representative immunofluorescence image showing oxidized DJ-1 protein (Cys106-SO_3,_ green fluorescence) in GBA1 K198E fibroblasts. (**F**) Representative fluorescence microscopy image showing oxidized DJ-1 protein (Cys106-SO_3,_ green fluorescence) in WT GBA1 fibroblast treated with ROT (1 μM). (**G**) Representative fluorescence microscopy image showing oxidized DJ-1 (Cys106-SO_3,_ green fluorescence) protein in GBA1 K198E fibroblast treated with ROT (1 μM). Nuclei were stained with Hoechst 33342 (blue fluorescence). (**H**) Quantitative analysis of oxidized DJ-1 protein (Cys106-SO_3_). Numbers in histograms represent positive cellular population for the tested marker. The histograms and photomicrographs represent one out of three independent experiments (n = 3). The data are presented as mean ± SD of three independent experiments (dots in bar). One-way ANOVA followed by Tukey’s test. Statistically significant differences: ** *p* < 0.01, *** *p* < 0.001; ns = not significance. Image magnification: 200×.

Next, we evaluated whether GBA1 K198E could induce oxidative stress through the generation of hydrogen peroxide (H_2_O_2_), a signaling molecule meanly generated by mitochondria [64]. Given that H_2_O_2_ is capable of specifically oxidizing the residue Cys106-SH (*sulfhydryl* group) of the DJ-1 protein into DJ-1 Cys106SO_3_ (*sulfonate* group) [65], we selected the protein DJ-1 as an oxidative stress sensor protein [66]. As shown in Figure 6A, WT fibroblasts showed no important oxidized DJ-1 protein, whereas GBA1 K198E fibroblasts endogenously displayed a significant oxidized DJ-1, i.e., an 11-fold increase (+900%) compared to WT fibroblasts. When WT and mutant fibroblasts were treated with ROT, WT and GBA1 K198E fibroblasts increased oxidized DJ-1 by +1700% and +59% (Figure 6B,C). Of note, there were no statistically significant differences in oxidized DJ-1 between WT and GBA1 K198E fibroblasts treated with ROT (Figure 6C). Similar observations were obtained by fluorescent microscopy analysis (Figure 6D–H). 

### 2.7. GBA1 K198E Fibroblasts Show Endogenously High Phosphorylated LRRK2 at Ser935 along with Phosphorylated α-Synuclein (α-Syn) at Pathological Residue Ser129

**Figure 7:**
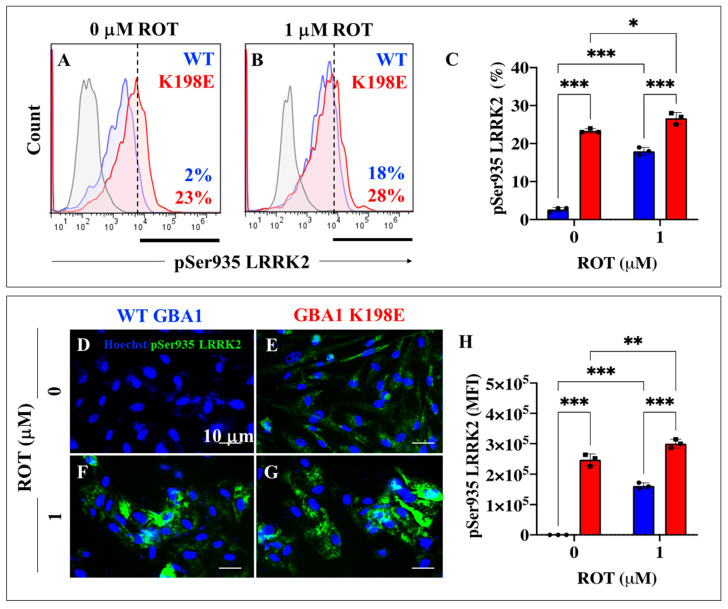
GBA1 K198E fibroblasts show an endogenously high percentage of phosphorylated LRRK2 at Ser935. (**A**) Representative flow cytometry histogram analysis showing phosphorylated LRRK2 at Ser935 protein in WT (blue curve) and GBA1 K198E fibroblasts (red curve). (**B**) Representative flow cytometry histogram analysis showing pSer935 LRRK2 protein in WT (blue curve) and GBA1 K198E fibroblasts (red curve) upon rotenone (ROT, 1 μM) exposure. (**C**) Quantitative analysis of pSer935 LRRK2 protein. (**D**) Representative immunofluorescence image showing pSer935 LRRK2 protein (green fluorescence) in WT GBA1 fibroblasts. (**E**) Representative immunofluorescence image showing pSer935 LRRK2 protein (green fluorescence) in GBA1 K198E fibroblasts. (**F**) Representative fluorescence microscopy image showing pSer935 LRRK2 protein (green fluorescence) in WT GBA1 fibroblast treated with ROT (1 μM). (**G**) Representative fluorescence microscopy image showing pSer935 LRRK2 protein (green fluorescence) protein in GBA1 K198E fibroblast treated with ROT (1 μM). Nuclei were stained with Hoechst 33342 (blue fluorescence). (**H**) Quantitative analysis of pSer935 LRRK2 protein. Numbers in histograms represent positive cellular population for the tested marker. The histograms and photomicrographs represent one out of three independent experiments (n = 3). The data are presented as mean ± SD of three independent experiments (dots in bar). One-way ANOVA followed by Tukey’s test. Statistically significant differences: * *p* <0.05, ** *p* < 0.01; *** *p* < 0.001. Image magnification: 200×.

Since H_2_O_2_ not only oxidized the stress sensor protein DJ-1 (as shown above) but also simultaneously induced the phosphorylation and activation of kinases such as LRRK2, a multifunctional protein involved in the phosphorylation of α-Syn at Ser129 [67], we evaluated whether GBA1 K198E fibroblasts could affect the phosphorylation status of LRRK2 kinase and α-Syn in mutant fibroblasts. Flow cytometry analysis shows that GBA1 K198E fibroblasts endogenously presented an increased phosphorylated LRRK2 by +1050% (Figure 7A,C) compared to WT fibroblasts (Figure 7C). While WT fibroblast treatment with ROT showed a dramatic increase in p-Ser935 LRRK2 by +800% (9-fold increase) compared to untreated WT fibroblasts, the GBA1 K198E exposed to ROT displayed a moderate increase in p-Ser935 LRRK2 by +22% compared to untreated mutant fibroblasts. We found a statistically significant tendency to increase p-Ser935 LRRK2 in mutant fibroblasts treated with ROT compared to untreated GBA1 K198E fibroblasts (Figure 7C). Similar data were obtained with fluorescence microscopy evaluation (Figure 7D–H). 

**Figure 8:**
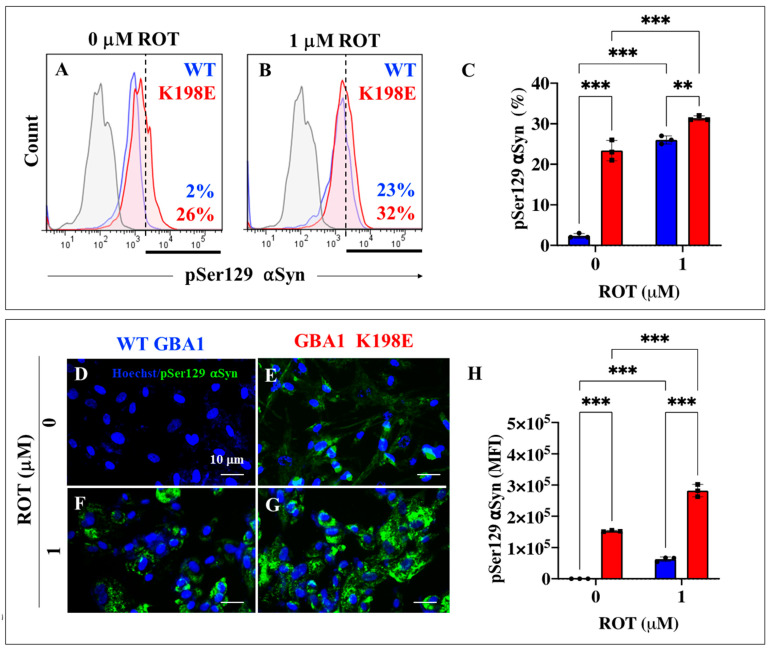
GBA1 K198E fibroblasts show an endogenously high percentage of phosphorylated α-Syn at Ser129. (**A**) Representative flow cytometry histogram analysis showing phosphorylated α-Syn at Ser129 protein in WT (blue curve) and GBA1 K198E fibroblasts (red curve). (**B**) Representative flow cytometry histogram analysis showing pSer129 α-Syn protein in WT (blue curve) and GBA1 K198E fibroblasts (red curve) upon rotenone (ROT, 1 μM) exposure. (**C**) Quantitative analysis of pSer129 α-Syn protein. (**D**) Representative immunofluorescence image showing pSer129 α-Syn protein (green fluorescence) in WT GBA1 fibroblasts. (**E**) Representative immunofluorescence image showing pSer129 α-Syn protein (green fluorescence) in GBA1 K198E fibroblasts. (**F**) Representative fluorescence microscopy image showing pSer129 α-Syn protein (green fluorescence) in WT GBA1 fibroblast treated with ROT (1 μM). (**G**) Representative fluorescence microscopy image showing pSer129 α-Syn protein (green fluorescence) in GBA1 K198E fibroblast treated with ROT (1 μM). Nuclei were stained with Hoechst 33342 (blue fluorescence). (**H**) Quantitative analysis of pSer129 α-Syn protein. Numbers in histograms represent positive cellular population for the tested marker. The histograms and photomicrographs represent one out of three independent experiments (n = 3). The data are presented as mean ± SD of three independent experiments (dots in bar). One-way ANOVA followed by Tukey’s test. Statistically significant differences: ** *p* < 0.01; *** *p* < 0.001. Image magnification: 200×.

In parallel, we evaluated whether phosphorylated α-Syn at pathological Ser129 was also present in GBA1 K198E fibroblasts. As shown in Figure 8, GBA1 K198E fibroblasts but not WT fibroblasts showed a significant endogenous high percentage of p-Ser129 α-Syn protein (+1200%, Figure 8A,C). Upon ROT exposure, WT fibroblasts increased p-Ser129 a-Syn by +800% (Figure 8B,C) compared to untreated WT fibroblasts (Figure 8A,C), whereas GBA1 K198E fibroblasts modestly augmented p-Ser129 α-Syn by +22% (Figure 8A–C). We also found a statistically significant increase in p-Ser129 α-Syn in mutant fibroblasts treated with ROT compared to untreated GBA1 K198E fibroblasts (Figure 8C). Similar data were obtained with fluorescence microscopy analysis (Figure 8D–H). 

### 2.8. GBA1 K198E Fibroblasts Show an Endogenously High Percentage of Cleaved Caspase 3 (CC3) Compared to WT Fibroblasts

**Figure 9:**
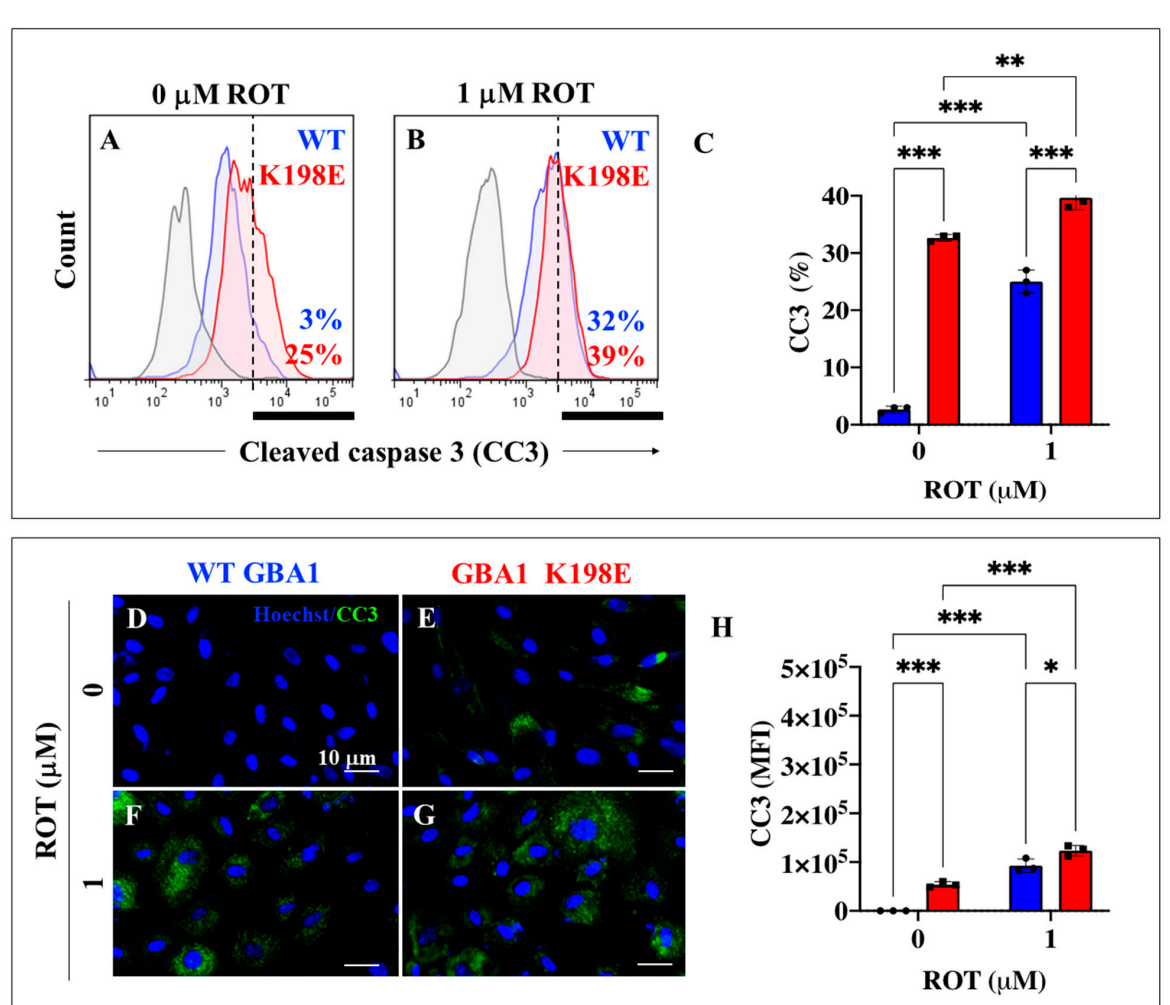
GBA1 K198E fibroblasts show endogenously high cleaved caspase 3 (CC3) compared to WT fibroblasts. (**A**) Representative flow cytometry histogram analysis showing cleaved caspase 3 (CC3) protein in WT (blue curve) and GBA1 K198E fibroblasts (red curve). (**B**) Representative flow cytometry histogram analysis showing CC3 protein in WT (blue curve) and GBA1 K198E fibroblasts (red curve) upon rotenone (ROT, 1 μM) exposure. (**C**) Quantitative analysis of CC3 protein. (**D**) Representative immunofluorescence image showing CC3 protein (green fluorescence) in WT GBA1 fibroblasts. (**E**) Representative immunofluorescence image showing CC3 protein (green fluorescence) in GBA1 K198E fibroblasts. (**F**) Representative fluorescence microscopy image showing CC3 protein (green fluorescence) in WT GBA1 fibroblast treated with ROT (1 μM). (**G**) Representative fluorescence microscopy image showing CC3 protein (green fluorescence) protein in GBA1 K198E fibroblast treated with ROT (1 μM). Nuclei were stained with Hoechst 33342 (blue fluorescence). (**H**) Quantitative analysis of CC3 protein. Numbers in histograms represent positive cellular population for the tested marker. The histograms and photomicrographs represent one out of three independent experiments (n = 3). The data are presented as mean ± SD of three independent experiments (dots in bar). One-way ANOVA followed by Tukey’s test. Statistically significant differences: * *p* < 0.05, ** *p* < 0.01; *** *p* < 0.001. Image magnification: 200×.

We further assessed whether the executor protein caspase 3 was active in GBA1 K198E fibroblasts. Like p-Ser935 LRRK2 and pSer129 α-Syn proteins, GBA1 K198E fibroblasts expressed an endogenously high percentage of CC3 by +733% (Figure 9A,C) compared to untreated WT fibroblasts (Figure 9C). When exposed to ROT, WT fibroblasts raised CC3 by +967% (Figure 9B,C), while GBA1 K198E fibroblasts modestly, but statistically significantly, elevated CC3 by +56% (Figure 9A–C) compared to treated mutant fibroblasts (Figure 9C). 

## 3. Discussion

In the present work, we demonstrate for the first time that the GBA1 K198E variant had a profound effect on the physiology of fibroblasts from a PD patient, and this effect resembles the effect of WT skin fibroblasts exposed to ROT. Indeed, we found that this variant dramatically inactivated the catalytic enzymatic activity of GCase (−70%) caused by a change in a positively charged (basic) a.a. lysine (K) for a negatively charged (acid) a.a. glutamic acid (E), thereby shifting the 3D structure of the enzyme [68], but increasing the protein expression levels (3.68-fold increase), most probably due to an enzymatic compensatory functional mechanism. These data imply that K198E alters enzyme functionality but it does interfere with its expression process. Theoretical analysis by in silico molecular docking shows that the K198E variant almost completely disables the binding catalytic pocket of the ligand–substrate (e.g., GlcSph). This observation suggests that K198E might partially or totally stop the enzymatic activity in mutant GCase (Table 1). Basically, the enzymatic anomaly has had far reaching physiological consequences for the normal metabolism of mutant fibroblasts, including deficiency in the autophagy–lysosome system, damage of the ∆Ψm, high OS, altered kinase activity (e.g., LRRK2), abnormal phosphorylation of presynaptic neuronal protein (e.g., p-Ser129 α-Syn), and diminished cell survival. It is concluded that the K198E mutation endogenously primes skin fibroblasts toward autophagy dysfunction, OS, and apoptosis. Several findings support this assumption. We show that the K198E mutation induced a deficiency in the autophagy–lysosomal pathway. This last observation is reinforced by an increase in the expression of Beclin-1, LC3-II, and LAMP-2 proteins, which are involved in the biogenesis of autophagosomes, i.e., initiation (e.g., Beclin-1), and elongation or autophagosome formation (e.g., LC3-II), and with the fusion of autophagic vacuoles with lysosomes (e.g., LAMP-2). Furthermore, the GBA1 K198E variant increases the accumulation of lysosomes in mutant but not WT fibroblasts. Taken together, these observations suggest that the K198E mutation complies with the notion that mutations in the *GBA1* gene lead to abnormal GCase enzymatic activity, which in turn provokes a deficient autophagosome–lysosomal–autolysosome system. Our data therefore support the notion that loss of GCase activity in the lysosome might be directly associated with deregulation of the autophagy process and PD pathology [35]. Interestingly, genetic alterations in other contributor genes in the autophagosome ensemble process (e.g., VPS35 [69]; LRRK2 [70]) or lysosome function (e.g., LRRK2) [71,72] have also been linked to PD pathophysiology [73,74]. These observations suggest that, for proper degradation and/or cleanness of unnecessary or dysfunctional neuronal cytosolic components, the autophagosome lysosome system has to be fine-tuned or highly regulated by a delicate balance between autophagosome ensemble, lysosome formation, and fusion of autophagosomes and lysosomes, resulting in a functional and efficient autolysosome vacuole [42,75]. In agreement with others [76], we reported the loss of ∆Ψm and OS in GBA1 K198E fibroblasts. Given that the GCase is imported into mitochondria and preserves complex I integrity and energy metabolism [59], it is reasonable to think that impaired GCase enzyme activity not only affects the lysosomal function to digest obsolete components of the cell itself but also impacts mitochondria to generate chemical energy. Therefore, GBA1/GCase has become critical in the pathophysiology of PD [77]. Not surprisingly, the GBA1 K198E phenotype was exacerbated by exposure to ROT, i.e., ROT-induced a high accumulation of lysosome and further decrease in ∆Ψm in mutant fibroblasts. Therefore, we conclude that the mechanistic link between GBA1 and PD lies in the interplay between GCase functions in the lysosome and mitochondria [38,78]. It is well known that Parkin protein plays an essential role in mitochondrial quality control of mitochondrial biogenesis, mitochondrial dynamics, and mitophagy [62]. Specifically, Parkin controls the lysosomal degradation of depolarized mitochondria through the mitophagy process [79]. Interestingly, it was observed that the expression of Parkin was comparable in naïve GBA1 K198E fibroblasts treated with ROT to WT fibroblasts treated with the same neurotoxin. These observations suggest that Parkin in mutant fibroblasts is irresponsive to further OS stimuli most probably because it is already exhausted by the permanent OS at which the cells are subjected, thereby being unable to increase its expression. 

Previous data have shown that pharmacological inhibition of GCase induces OS in SHSY-5Y cells [76]. Likewise, we found that GBA1 K198E fibroblasts show oxidation of stress sensor DJ-1 at residue Cys106 (−SO_3_) as evidence of OS via specific interaction with H_2_O_2_ [65], produced most probably from the malfunctional mitochondria. This assumption is reinforced by the observation that ROT, a mitochondrial complex I inhibitor, notably increases the detection of DJ-1 Cys106-SO_3_ in both WT and GBA1 K198E fibroblasts. Taken together, these observations suggest that the source of the oxidizing agent H_2_O_2_ might be the mitochondrial complex I. However, investigation is needed to gain furhter insight on this issue. Interestingly, there were no statistically significant differences in oxidized DJ-1 between WT and GBA1 K198E fibroblasts treated with ROT. These findings imply that GBA1 K198E fibroblasts are permanently in the OS state similar to WT fibroblasts exposed to ROT. Given that H_2_O_2_ can also operate as an intracellular messenger [80], it is then capable of inducing the activation of other signaling molecules involving kinases [64]. Effectively, we found that LRRK2 kinase is abnormally phosphorylated at residue Ser935 in GBA1 K198E fibroblasts as well as in WT fibroblasts treated with ROT. In accordance with this finding, H2O2 might activate LRRK2 kinase activity by directly enhancing its autophosphorylation, e.g., at Tyr1967 [81], Ser2032, and Tyr2035 [82,83], or indirectly, via phosphorylation of Ser910 and Ser935 via the inhibitor of nuclear factor-κB (IκB) kinase (IKK) complex [84]. Whatever the mechanism of phosphorylation of LRRK2 might be, it is endogenously highly phosphorylated in GBA1 K198E fibroblasts. In turn, we also simultaneously found abnormally phosphorylated α-Syn at pathological Ser129 in both GBA1 K198E fibroblasts and WT fibroblasts treated with ROT. These observations suggest that, once active, the LRRK2 kinase phosphorylates α-Syn at Ser129 [67], which is the major component of pathological deposits in PD [85,86]. It is worth mentioning that the inhibitor LRRK2 kinase PF-06447475 almost completely abolished the p-Ser129-α-Syn in ROT-induced OS models [87,88], but also LRRK2 knockout confers resistance against ROT-induced OS, mitochondrial damage, and apoptosis [89]. Taken together these findings support the notion that H2O2 indirectly phosphorylates LRRK2, which in turn phosphorylates α-Syn (p-Ser129 α-Syn) in GBA1 K198E fibroblasts. 

Cleaved caspase 3 (CC3) has amply been used as a final effector in the apoptotic cell death of DAergic neurons in PD [90]. In line with this, we detected a significantly higher percentage of CC3-positive in GBA1 K198E fibroblasts and in WT fibroblasts exposed to ROT than in controls. Taken together, these observations reinforced the notion that several molecular events are intrinsically active in GBA1 K198E fibroblasts, which might contribute to their deterioration and final demise. 

## 4. Materials and Methods

### 4.1. Human Dermal Fibroblast Culture

Fibroblasts were obtained from skin biopsies of one PD patient carrying a heterozygous GBA1 mutation (K198E, Tissue Bank Code (TBC) # COP0826, male, age at onset 33 years, age at sampling 58 years old) and one healthy control (TBC # 10624, male, age at sampling 58 years old) matched for age and gender. This study was approved by the Ethics Committee of Research from “Sede de Investigación Universitaria-SIU” (approval code 19-10-845). To isolate dermal fibroblasts, 4 mm skin biopsies of the donors were obtained using a biopsy punch. Skin tissue was cut up into small pieces (<1 mm), placed into gelatin-coated 6-well dishes (Costar Corning Incorporated), and left to dry at 37 °C until attachment. Once the explants were attached to the plate, culture medium (Dulbecco’s Modified Eagle’s Medium; DMEM, cat #D0819, Sigma, Saint Louis, MO, USA) supplemented with 10% fetal bovine serum (FBS, cat #CVFSVF00-01, Eurobio Scientific, Paris, France) and 1% penicillin/streptomycin (P/S; Gibco) (10% DMEM) was carefully added, and the plates were incubated at 37 °C in a humidified atmosphere with 5% CO_2_. When cells sprouted from the explants, after one week in culture, the plates were washed with PBS to eliminate non-adherent cells and surplus explants, and new 10% DMEM culture medium was added. The culture medium was then replaced every 3–4 days. When sprouted cells reached 80% confluency, subculturing was performed for cell expansion.

### 4.2. Analysis of Cells 

#### 4.2.1. Assay Protocol

WT and GBA1 K198E fibroblasts were cultured in DMEM with low glucose (1000 mg/L) plus 10% fetal bovine serum (FBS). For in vitro stimulation and inhibition of autophagy, cells were left untreated or treated with rapamycin (RAP; 10 nM, Cat# 53123-88-9, Sigma-Aldrich, St. Louis, MO, USA) and bafilomycin A1 (BAF; 10 nM, Cat No. B1793, Sigma-Aldrich, St. Louis, USA) for 24 h at 37 °C. For in vitro inhibition of mitochondrial activity, cells were left untreated or treated with rotenone (ROT, 1 μM) for 24 h at 37 °C.

#### 4.2.2. Glucocerebrosidase (GCase) Activity Assay 

Cellular GCase activity was determined using the Beta-Glucosidase Assay Kit (Abcam, Boston, MA, USA, Cat. ab272521) according to the manufacturer’s recommendations with minor modifications. Briefly, WT and K198E untreated fibroblast cell lysates were incubated for 24 h at 37 °C with p-nitrophenyl-α-d-glucopyranoside, which was hydrolyzed specifically by β-glucosidase into a yellow-colored product (maximal absorbance at 405 nm). The rate of the reaction was directly proportional to the enzyme activity. 

#### 4.2.3. Autophagy Assay

The autophagy assay was performed according to the manufacturer’s recommendation (cat #MAK138, Sigma-Aldrich, Saint Louis, MO, USA). Briefly, cells under different treatments were incubated with 1X stain reagent for 20 min. Then, the fluorescence intensity (ex 360/em 520 nm) was measured using a BD LSRFortessa II flow cytometer (BD Biosciences, Franklin Lakes, NJ, USA). Twenty-thousand events were acquired, and the acquisition analysis was performed using FlowJo 7.6.2 Data Analysis Software (BD Biosciences, Franklin Lakes, NJ, USA). For fluorescence microscopy analysis, cells were incubated with 1X stain reagent for 20 min and nuclei were stained with (0.5 μM) Hoechst 33342 (Life Technologies, Carlsbad, CA, USA), and fluorescence microscopy photographs were taken using a Zeiss Axio Vert.A1 inverted Fluorescence Microscope equipped with a Zeiss AxioCam Cm1. 

### 4.3. Flow Cytometry and Fluorescent Microscopy Immunofluorescence

After each treatment, cells (1 × 10^5^) were carefully detached and fixed in 80% ethanol and stored at 20 °C overnight. Then, cells were washed with PBS and permeabilized with 0.2% triton X-100 (Cat# 93443, Sigma-Aldrich, St. Louis, MO, USA) plus 1.5% bovine serum albumin (BSA, Cat# A9418, Sigma-Aldrich, St. Louis, MO, USA) in phosphate-buffered solution (PBS) for 30 min. Then, cells were washed and incubated with primary antibodies (1:200; diluted in PBS containing 0.1% BSA) against GCase (1:500, Cat. #G4171, Sigma), LC3-II (cat #NB100-2220, Novus Biologicals, Englewood, CO, USA), Beclin1 (proteintech, cat #66665-1-Ig, USA), LAMP-2 (Sigma-Aldrich, cat #MABC1766, St. Louis, MO, USA), Parkin (cat #30130, Santa Cruz Biotech, Santa Cruz, CA, USA), p-(S935)-LRRK2 (Abcam cat #AB133450; Boston, MA, USA), α-synuclein (pS129; Abcam cat #AB51253; Boston, MA, USA), oxidized DJ-1 (1:500; ox (Cys106) DJ-1; spanning residue C106 of human PARK7/DJ1; oxidized to produce cysteine sulfonic (SO3) acid; Abcam cat #AB169520; Boston, MA, USA) and cleaved caspase 3, (CC3; 1:250; cat# AB3623, Millipore, Merck, Darmstadt, Germany) overnight at 4 °C. After exhaustive rinsing, we incubated the cells with secondary fluorescent antibodies (DyLight 488 horse anti-rabbit and mouse antibodies, cats DI 1094 and DI 2488, Vector Laboratories, Newark, NJ, USA) at 1:500. Finally, cells were washed and re-suspended in PBS for analysis on a BD LSRFortessa II flow cytometer (BD Biosciences, Franklin Lakes, NJ, USA). Twenty-thousand events were acquired, and the acquisition analysis was performed using FlowJo 7.6.2 Data Analysis Software (BD Biosciences, Franklin Lakes, NJ, USA). For fluorescence microscopy analysis, attached cells were fixed in 80% ethanol and incubated with primary and secondary antibodies as described above. Nuclei were stained with (0.5 μM) Hoechst 33342 and fluorescence microscopy photographs were taken using a Zeiss Axio Vert.A1 Fluorescence Microscope equipped with a Zeiss AxioCam Cm1. 

### 4.4. Characterization of Lysosomal Complexity

To analyze lysosomal complexity, cells were incubated with the cell-permeable, nonfixable, green, fluorescent dye LysoTracker Green DND-26 (50 nM, cat #L7526, Thermo Fisher Scientific, Waltham, MA, USA) for 30 min at 37 °C. Then, the cells were washed, and LysoTracker fluorescence was determined by flow cytometry using a BD LSRFortessa II flow cytometer (BD Biosciences, Franklin Lakes, NJ, USA) or fluorescent microscopy using a Zeiss Axio Vert.A1 Fluorescence Microscope equipped with a Zeiss AxioCam Cm1. The experiment was conducted three times, and 20,000 events were acquired for analysis. Flow cytometry analysis for LysoTracker was performed by selecting, in the FL-1 channel, all cells with LysoTracker reactivity (>99%). Quantitative data and figures were obtained using FlowJo 7.6.2 Data Analysis Software (BD Biosciences, Franklin Lakes, NJ, USA).

### 4.5. Analysis of Mitochondrial Membrane Potential (ΔΨm) 

Assessment of the ΔΨm was performed according to Ref. [91]. We incubated cells for 20 min at RT in the dark with a deep red MitoTracker (20 nM final concentration) compound (Thermo Scientific, cat# M22426, Netherlands, Europe). Cells were analyzed with flow cytometry using a BD LSRFortessa II flow cytometer (BD Biosciences, Franklin Lakes, NJ, USA) or fluorescent microscopy using a Zeiss Axio Vert.A1 Fluorescence Microscope equipped with a Zeiss AxioCam Cm1. The experiment was conducted three times, and 20,000 events were acquired for analysis. Quantitative data and figures were obtained using FlowJo 7.6.2 Data Analysis Software.

### 4.6. Molecular Docking 

To enable the 3D structure of GBA1, the AlphaFold2.ipynb program (https://colab.research.google.com/github/sokrypton/ColabFold/blob/main/AlphaFold2.ipynb, accessed on 8 June 2024) was loaded with the WT (K198K) and GBA1 mutant K198E amino acid sequences (Uniprot ID: P04062; GBA1_HUMAN; mature protein 497 a.a.; https://www.uniprot.org/uniprotkb/P04062/entry#function, accessed on 8 June 2024) to model the structure of WT and K198E mutated protein glucocerobrosidase (GCase), respectively. The blind molecular docking was performed with CB-Dock version 2 (a cavity detection-guided protein–ligand blind docking web server that uses Autodock Vina (version 1.1.2, Scripps Research Institute, La Jolla, USA; https://cadd.labshare.cn/cb-dock2/php/blinddock.php, accessed on 8 June 2024). The SDF structure files of glucosylsphingosine (GlcSph, compound CID 5280570) and conduritol-B-epoxide (CBE, compound CID 136345) were downloaded from PubChem (https://pubchem.ncbi.nlm.nih.gov/, accessed on 8 June 2024). The molecular blind docking was performed by uploading the 3D structure PDB file of GCase into the server with the SDF file of each compound. For analysis, we selected the docking poses with the strongest Vina score in the catalytical pocket. The generated PDB files of the molecular docking of each compound were visualized with the CB-Dock2 interphase.

### 4.7. Data Analysis

In this experimental design, two vials of fibroblast were thawed (WT GBA1 and GBA1 K198E), cultured, and the cell suspension was pipetted at a standardized cellular density of 2 × 10^4^ cells/cm^2^ into different wells of a 24- or 6-well plate. Cells (i.e., the biological and observational units) [92] were randomized to wells by simple randomization (sampling without replacement method), and then wells (i.e., the experimental units) were randomized to treatments by a similar method. Experiments were performed on three independent occasions (n = 3) blind to the experimenter and/or flow cytometer analyst [92]. The data from the three repetitions, i.e., independent experiments, were averaged, and representative flow cytometry density or histogram plots from the three independent experiments were selected for illustrative purposes, whereas the bars in the quantification figures represent the mean ± SD and the three black dots show the data point of each experimental repetition. Based on the assumptions that the experimental unit (i.e., the well) data comply with the independence of observations, the dependent variable is normally distributed in each treatment group (Shapiro–Wilk test), and there is homogeneity of variances (Levene’s test), where the statistical significance is determined by a one-way analysis of variance (ANOVA) followed by Tukey’s post hoc comparison calculated with GraphPad Prism 5.0 software. Differences between groups were only deemed significant with a p-value of 0.05 (*), 0.01 (**), and 0.001 (***). All data are presented as the mean ± S.D.

## 5. Conclusions

**Figure 10:**
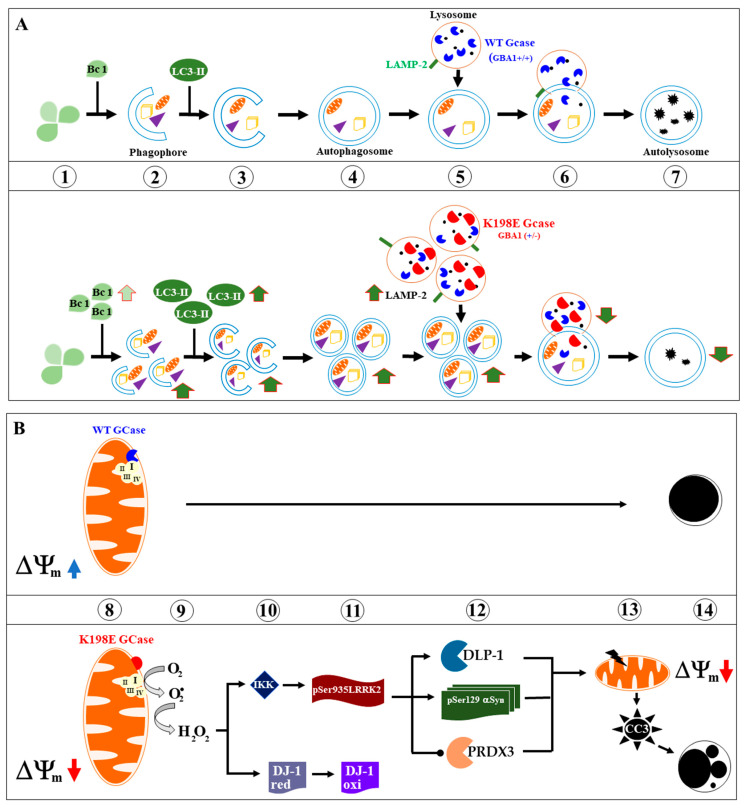
Schematic representation of the effect of K198E GCase on the autophagy–lysosomal pathway and apoptosis cell death in skin fibroblasts. (**A**) *Autophagy–lysosomal pathway and K198E GCase.* In WT GBA1 fibroblasts, the autophagy process begins with the ensemble of the ULK1 complex (unc-51-like kinase, ULK; autophagy-related protein 13, ATG13; RB1-inducible coiled-coil protein 1, FIP200; ATG101) (**1**), which then triggers nucleation of the phagophore (**2**) by phosphorylating components of the class III PI3K complex, involving Class IIIPI3K, vacuolar protein sorting 34 (VPS34) and Beclin 1 (Bc 1), among other proteins. These actions lead to the attachment of the microtubule-associated protein light chain 3 (LC3-II) to the phagophore (**3**), which further expand and form a sealed double-membrane, forming the autophagosome (**4**). This last vacuole, helped by lysosomal-associated membrane protein (LAMP-2, **5**), fused with the lysosome (**6**) to form the autolysosome (**7**), where unwanted cytosolic material (damaged mitochondria, protein aggregates, GlcCer (black dots)) is eliminated and recycled. On the other hand, enzymatic alteration of GCase mainly by genetic mutations (e.g., K198E) in at least one of the alleles of GBA1 almost leads to the undigested substrate GlcCer. As a result, lysosomes accumulate, thereby affecting the production line of autophagosomes, and autolysosomes. Indeed, heterozygous K198E GCase induces abnormal upregulation of protein Beclin 1, LC3-II and LAMP-2, and provokes an abnormally high accumulation of autophagosomes, lysosomes, and autolysosomes. Overall, K198E GCase provokes a highly deficient autophagy–lysosomal pathway in skin fibroblasts. (**B**) *Apoptosis pathway and K198E GCase.* In parallel, WT GCase interacts the mitochondrial respiratory component complex I (**8**, upper panel), thereby preserving energy metabolism (e.g., high ∆Ψm) and mitochondrial and nuclei integrity (**14**, upper panel). On the contrary, malfunction of mitochondrial Complex I due to improper interactions with K198E GCase (**8**, lower panel) allows electrons to leak, which are taken by molecular dioxygen (O2). Reduction of oxygen ends up in the formation of anion superoxide radicals (O2.-), which then dismutate into H2O2 (**9**). As a messenger molecule, H2O2 oxidized DJ-1Cys106-SOH (DJ-1red, **10**) into DJ-1Cys106-SO3 (-*sulfonic* group, DJ-1oxi, **11**) and induces the activation of the IKK complex (**10**), which phosphorylates LRRK2 at residue Ser935 (**11**). Phosphorylated LRRK2 kinase phosphorylates in turn the following three main targets: DLP-1 (dynamin-like protein), αSyn at residue Ser129, and PRDX3 (**12**). These three proteins might induce or contribute to mitochondria depolarization (e.g., low ∆Ψm), thereby inducing activation of caspase 3 (CASP3) into cleaved caspase 3 (CC3, **13**). Lastly, CC3 induces the fragmentation of nuclei (**14**). All these markers constitute typical signs of apoptosis (**8**–**14**).

In the present work, we characterize for the first time the cellular and molecular phenotype of skin fibroblasts from a PD patient bearing the mutation GBA1 K198E, which is a specific variant to the Colombian population [36]. We conclude that the GBA1 K198E mutation endogenously primes PD skin fibroblasts toward autophagy dysfunction, OS, and apoptosis. Furthermore, our findings suggest that the GBA1 K198E fibroblasts are biochemically and molecularly equivalent to the response of WT GBA1 fibroblasts exposed to ROT. Given that GCase is localized in lysosomes and mitochondria, a proposed mechanism by which heterozygous GBA1 K198E mutation causes deterioration in fibroblasts (or in neuronal cells) is illustrated in Figure 10. As validation of this model, a similar mechanism of action affecting autophagy and apoptosis has been shown for ROT, which selectively blocks mitochondrial complex I and lysosomal GCase [39]. Taken together, these observations suggest that GBA1 K198E mutations are amenable to pharmacological treatment [93]. Therefore, further investigation is warranted on this issue. Our investigation further validates primary skin fibroblasts as a useful PD model and their ability to serve as substitute cells of neuronal tissue [94,95]. 

## Figures and Tables

**Table 1:** In silico molecular docking analysis of glucocerebrosidase (GCase), glucosyl sphingosine (GlcSph), and conduritol-B-epoxide (CBE).

Submitted Protein *	Submitted Ligand **	Vina Score ***	Cavity Volume (Å^3^)	Center (x, y, z)	Docking Size (x, y, z)	Contact Residues
**WT** **GCase**	Glucosylsphingosine CID: 5280570	**−6.1**	447	9, −12, 0	28, 28, 28	Chain A: ASP127 TRP179 ASN234 **GLU235** TYR244 PRO245 PHE246 TYR313 LEU314 **GLU340** GLY344 **SER345** LYS346 PHE347 GLU349 GLN350 **SER351** TRP381 ARG395 ASN396 PHE397 VAL398
**K198E GCase**	Glucosylsphingosine CID: 5280570	-	-	-	-	-
**WT** **GCase**	Conduritol B epoxide CID: 136345	**−5.7**	447	9, −12, 0	16, 16, 16	Chain A: **ASP127** PHE128 TRP179 ASN234 **GLU235** TYR244 PHE246 GLN284 TYR313 **GLU340** CYS342 SER345 TRP381 ASN396 VAL398
**K198E** **GCase**	Conduritol-B-epoxide CID: 136345	-	-	-	-	-

* According to RCSB Protein Data Base (https://www.rcsb.org/ accessed on 30 May 2024). ** According to PubChem database (https://pubchem.ncbi.nlm.nih.gov/ accessed on 30 May 2024). *** According to CB-dock2: An accurate protein–ligand bind docking tool (https://cadd.labshare.cn/cb-dock2/php/index.php/ accessed on 30 May 2024). **Bold** represents the amino acid of GCase involved in the conventional hydrogen bonding with Glucosylsphingosine, and conduritol-B-epoxide. **Bold blue** letters represent the amino acid of the enzymatic active pocket of GCase.

## Data Availability

All relevant data are within the manuscript.

## References

[1] Parkinson J. (2002). An Essay on the Shaking Palsy. J. Neuropsychiatry Clin. Neurosci..

[2] Dorsey E.R., Sherer T., Okun M.S., Bloemd B.R. (2018). The Emerging Evidence of the Parkinson Pandemic. J. Parkinson’s Dis..

[3] Dorsey E.R., Elbaz A., Nichols E., Abbasi N., Abd-Allah F., Abdelalim A., Adsuar J.C., Ansha M.G., Brayne C., Choi J.-Y.J. (2018). Global, Regional, and National Burden of Parkinson’s Disease, 1990–2016: A Systematic Analysis for the Global Burden of Disease Study 2016. Lancet Neurol..

[4] Ou Z., Pan J., Tang S., Duan D., Yu D., Nong H., Wang Z. (2021). Global Trends in the Incidence, Prevalence, and Years Lived with Disability of Parkinson’s Disease in 204 Countries/Territories from 1990 to 2019. Front. Public Health.

[5] Váradi C. (2020). Clinical Features of Parkinson’s Disease: The Evolution of Critical Symptoms. Biology.

[6] Lysia S. (1996). Forno Neuropathology of Parkinson’s Disease. J. Neuropathol. Exp. Neurol..

[7] Goedert M., Spillantini M.G., Del Tredici K., Braak H. (2013). 100 Years of Lewy Pathology. Nat. Rev. Neurol..

[8] Engelhardt E., Gomes M.d.M. (2017). Lewy and His Inclusion Bodies: Discovery and Rejection. Dement. Neuropsychol..

[9] Spillantini M.G., Crowther R.A., Jakes R., Hasegawa M., Goedert M. (1998). α-Synuclein in Filamentous Inclusions of Lewy Bodies from Parkinson’s Disease and Dementia with Lewy Bodies. Proc. Natl. Acad. Sci. USA.

[10] Islam M.S., Azim F., Saju H., Zargaran A., Shirzad M., Kamal M., Fatema K., Rehman S., Azad M.A.M., Ebrahimi-Barough S. (2021). Pesticides and Parkinson’s Disease: Current and Future Perspective. J. Chem. Neuroanat..

[11] Bloem B.R., Boonstra T.A. (2023). The Inadequacy of Current Pesticide Regulations for Protecting Brain Health: The Case of Glyphosate and Parkinson’s Disease. Lancet Planet. Health.

[12] Bogers J.S., Bloem B.R., Den Heijer J.M. (2023). The Etiology of Parkinson’s Disease: New Perspectives from Gene-Environment Interactions. J. Parkinson’s Dis..

[13] Lim S.-Y., Klein C. (2024). Parkinson’s Disease Is Predominantly a Genetic Disease. J. Parkinson’s Dis..

[14] Pang S.Y.-Y., Ho P.W.-L., Liu H.-F., Leung C.-T., Li L., Chang E.E.S., Ramsden D.B., Ho S.-L. (2019). The Interplay of Aging, Genetics and Environmental Factors in the Pathogenesis of Parkinson’s Disease. Transl. Neurodegener..

[15] Jia F., Fellner A., Kumar K.R. (2022). Monogenic Parkinson’s Disease: Genotype, Phenotype, Pathophysiology, and Genetic Testing. Genes.

[16] Day J.O., Mullin S. (2021). The Genetics of Parkinson’s Disease and Implications for Clinical Practice. Genes.

[17] Matsui H., Takahashi R. (2024). Current Trends in Basic Research on Parkinson’s Disease: From Mitochondria, Lysosome to α-Synuclein. J. Neural. Transm..

[18] Jankovic J., Tan E.K. (2020). Parkinson’s Disease: Etiopathogenesis and Treatment. J. Neurol. Neurosurg. Psychiatry.

[19] Jiang P., Dickson D.W. (2018). Parkinson’s Disease: Experimental Models and Reality. Acta Neuropathol..

[20] Airavaara M., Parkkinen I., Konovalova J., Albert K., Chmielarz P., Domanskyi A. (2020). Back and to the Future: From Neurotoxin-Induced to Human Parkinson’s Disease Models. Curr. Protoc. Neurosci..

[21] Lawana V., Cannon J.R. (2020). Rotenone Neurotoxicity: Relevance to Parkinson’s Disease. Advances in Neurotoxicology.

[22] Teves J.M.Y., Bhargava V., Kirwan K.R., Corenblum M.J., Justiniano R., Wondrak G.T., Anandhan A., Flores A.J., Schipper D.A., Khalpey Z. (2018). Parkinson’s Disease Skin Fibroblasts Display Signature Alterations in Growth, Redox Homeostasis, Mitochondrial Function, and Autophagy. Front. Neurosci..

[23] Deus C.M., Pereira S.P., Cunha-Oliveira T., Pereira F.B., Raimundo N., Oliveira P.J. (2020). Mitochondrial Remodeling in Human Skin Fibroblasts from Sporadic Male Parkinson’s Disease Patients Uncovers Metabolic and Mitochondrial Bioenergetic Defects. Biochim. Biophys. Acta (BBA)-Mol. Basis Dis..

[24] Horowitz M., Wilder S., Horowitz Z., Reiner O., Gelbart T., Beutler E. (1989). The Human Glucocerebrosidase Gene and Pseudogene: Structure and Evolution. Genomics.

[25] Dvir H., Harel M., McCarthy A.A., Toker L., Silman I., Futerman A.H., Sussman J.L. (2003). X-ray Structure of Human Acid-β-glucosidase, the Defective Enzyme in Gaucher Disease. EMBO Rep..

[26] Reczek D., Schwake M., Schröder J., Hughes H., Blanz J., Jin X., Brondyk W., Van Patten S., Edmunds T., Saftig P. (2007). LIMP-2 Is a Receptor for Lysosomal Mannose-6-Phosphate-Independent Targeting of β-Glucocerebrosidase. Cell.

[27] Gonzalez A., Valeiras M., Sidransky E., Tayebi N. (2014). Lysosomal Integral Membrane Protein-2: A New Player in Lysosome-Related Pathology. Mol. Genet. Metab..

[28] Tamargo R.J., Velayati A., Goldin E., Sidransky E. (2012). The Role of Saposin C in Gaucher Disease. Mol. Genet. Metab..

[29] Atrian S., López-Viñas E., Gómez-Puertas P., Chabás A., Vilageliu L., Grinberg D. (2008). An Evolutionary and Structure-based Docking Model for Glucocerebrosidase–Saposin C and Glucocerebrosidase–Substrate Interactions—Relevance for Gaucher Disease. Proteins Struct. Funct. Bioinform..

[30] Kompoliti K., Verhagen L. (2010). Encyclopedia of Movement Disorders.

[31] Vieira S.R.L., Schapira A.H.V. (2021). Glucocerebrosidase Mutations: A Paradigm for Neurodegeneration Pathways. Free. Radic. Biol. Med..

[32] Vieira S.R.L., Schapira A.H.V. (2022). Glucocerebrosidase Mutations and Parkinson Disease. J. Neural. Transm..

[33] Migdalska-Richards A., Schapira A.H.V. (2016). The Relationship between Glucocerebrosidase Mutations and Parkinson Disease. J. Neurochem..

[34] Menozzi E., Schapira A.H.V. (2021). Exploring the Genotype–Phenotype Correlation in GBA-Parkinson Disease: Clinical Aspects, Biomarkers, and Potential Modifiers. Front. Neurol..

[35] Onal G., Yalçın-Çakmaklı G., Özçelik C.E., Boussaad I., Şeker U.Ö.Ş., Fernandes H.J.R., Demir H., Krüger R., Elibol B., Dökmeci S. (2024). Variant-Specific Effects of GBA1 Mutations on Dopaminergic Neuron Proteostasis. J. Neurochem..

[36] Velez-Pardo C., Lorenzo-Betancor O., Jimenez-Del-Rio M., Moreno S., Lopera F., Cornejo-Olivas M., Torres L., Inca-Martinez M., Mazzetti P., Cosentino C. (2019). The Distribution and Risk Effect of GBA Variants in a Large Cohort of PD Patients from Colombia and Peru. Park. Relat. Disord..

[37] Orvisky E., Park J.K., Parker A., Walker J.M., Martin B.M., Stubblefield B.K., Uyama E., Tayebi N., Sidransky E. (2002). The Identification of Eight Novel Glucocerebrosidase (GBA) Mutations in Patients with Gaucher Disease. Hum. Mutat..

[38] Chatterjee D., Krainc D. (2023). Mechanisms of Glucocerebrosidase Dysfunction in Parkinson’s Disease. J. Mol. Biol..

[39] Perez-Abshana L.P., Mendivil-Perez M., Velez-Pardo C., Jimenez-Del-Rio M. (2023). Rotenone Blocks the Glucocerebrosidase Enzyme and Induces the Accumulation of Lysosomes and Autophagolysosomes Independently of LRRK2 Kinase in HEK-293 Cells. Int. J. Mol. Sci..

[40] dos Santos J.C.C., Mano G.B.C., da Cunha Barreto-Vianna A.R., Garcia T.F.M., de Vasconcelos A.V., Sá C.S.G., de Souza Santana S.L., Farias A.G.P., Seimaru B., Lima M.P.P. (2024). The Molecular Impact of Glucosylceramidase Beta 1 (Gba1) in Parkinson’s Disease: A New Genetic State of the Art. Mol. Neurobiol..

[41] Zhang J. (2015). Teaching the Basics of Autophagy and Mitophagy to Redox Biologists—Mechanisms and Experimental Approaches. Redox. Biol..

[42] Lőrincz P., Juhász G. (2020). Autophagosome-Lysosome Fusion. J. Mol. Biol..

[43] Wipperman M.F., Montrose D.C., Gotto A.M., Hajjar D.P. (2019). Mammalian Target of Rapamycin. Am. J. Pathol..

[44] Lippai M., Szatmári Z. (2017). Autophagy—From Molecular Mechanisms to Clinical Relevance. Cell. Biol. Toxicol..

[45] Dikic I., Elazar Z. (2018). Mechanism and Medical Implications of Mammalian Autophagy. Nat. Rev. Mol. Cell Biol..

[46] Galluzzi L., Vitale I., Aaronson S.A., Abrams J.M., Adam D., Agostinis P., Alnemri E.S., Altucci L., Amelio I., Andrews D.W. (2018). Molecular Mechanisms of Cell Death: Recommendations of the Nomenclature Committee on Cell Death 2018. Cell Death Differ..

[47] Collins L.M., Drouin-Ouellet J., Kuan W.-L., Cox T., Barker R.A. (2017). Dermal Fibroblasts from Patients with Parkinson’s Disease Have Normal GCase Activity and Autophagy Compared to Patients with PD and GBA Mutations. F1000Research.

[48] Grace M.E., Newman K.M., Scheinker V., Berg-Fussman A., Grabowski G.A. (1994). Analysis of Human Acid Beta-Glucosidase by Site-Directed Mutagenesis and Heterologous Expression. J. Biol. Chem..

[49] Sawkar A.R., Adamski-Werner S.L., Cheng W.-C., Wong C.-H., Beutler E., Zimmer K.-P., Kelly J.W. (2005). Gaucher Disease-Associated Glucocerebrosidases Show Mutation-Dependent Chemical Chaperoning Profiles. Chem. Biol..

[50] Liou B., Kazimierczuk A., Zhang M., Scott C.R., Hegde R.S., Grabowski G.A. (2006). Analyses of Variant Acid β-Glucosidases. J. Biol. Chem..

[51] Premkumar L., Sawkar A.R., Boldin-Adamsky S., Toker L., Silman I., Kelly J.W., Futerman A.H., Sussman J.L. (2005). X-ray Structure of Human Acid-β-Glucosidase Covalently Bound to Conduritol-B-Epoxide. J. Biol. Chem..

[52] Pradas E., Martinez-Vicente M. (2023). The Consequences of GBA Deficiency in the Autophagy–Lysosome System in Parkinson’s Disease Associated with GBA. Cells.

[53] Querfurth H., Lee H.-K. (2021). Mammalian/Mechanistic Target of Rapamycin (MTOR) Complexes in Neurodegeneration. Mol. Neurodegener..

[54] Wang R., Wang J., Hassan A., Lee C.-H., Xie X.-S., Li X. (2021). Molecular Basis of V-ATPase Inhibition by Bafilomycin A1. Nat. Commun..

[55] Tran S., Fairlie W.D., Lee E.F. (2021). BECLIN1: Protein Structure, Function and Regulation. Cells.

[56] Tanida I., Ueno T., Kominami E. (2004). LC3 Conjugation System in Mammalian Autophagy. Int. J. Biochem. Cell Biol..

[57] Eskelinen E.-L., Illert A.L., Tanaka Y., Schwarzmann G., Blanz J., von Figura K., Saftig P. (2002). Role of LAMP-2 in Lysosome Biogenesis and Autophagy. Mol. Biol. Cell.

[58] van Meel E., Bos E., van der Lienden M.J.C., Overkleeft H.S., van Kasteren S.I., Koster A.J., Aerts J.M.F.G. (2019). Localization of Active Endogenous and Exogenous Β-glucocerebrosidase by Correlative Light-electron Microscopy in Human Fibroblasts. Traffic.

[59] Baden P., Perez M.J., Raji H., Bertoli F., Kalb S., Illescas M., Spanos F., Giuliano C., Calogero A.M., Oldrati M. (2023). Glucocerebrosidase Is Imported into Mitochondria and Preserves Complex I Integrity and Energy Metabolism. Nat. Commun..

[60] Read A.D., Bentley R.E.T., Archer S.L., Dunham-Snary K.J. (2021). Mitochondrial Iron–Sulfur Clusters: Structure, Function, and an Emerging Role in Vascular Biology. Redox. Biol..

[61] Pereira C.S., Teixeira M.H., Russell D.A., Hirst J., Arantes G.M. (2023). Mechanism of Rotenone Binding to Respiratory Complex I Depends on Ligand Flexibility. Sci. Rep..

[62] Wang X.-L., Feng S.-T., Wang Z.-Z., Yuan Y.-H., Chen N.-H., Zhang Y. (2021). Parkin, an E3 Ubiquitin Ligase, Plays an Essential Role in Mitochondrial Quality Control in Parkinson’s Disease. Cell. Mol. Neurobiol..

[63] Araiso Y., Endo T. (2022). Structural Overview of the Translocase of the Mitochondrial Outer Membrane Complex. Biophys. Physicobiol..

[64] Di Marzo N., Chisci E., Giovannoni R. (2018). The Role of Hydrogen Peroxide in Redox-Dependent Signaling: Homeostatic and Pathological Responses in Mammalian Cells. Cells.

[65] Kinumi T., Kimata J., Taira T., Ariga H., Niki E. (2004). Cysteine-106 of DJ-1 Is the Most Sensitive Cysteine Residue to Hydrogen Peroxide-Mediated Oxidation in Vivo in Human Umbilical Vein Endothelial Cells. Biochem. Biophys. Res. Commun..

[66] Yoshida Y., Saito Y. (2014). Oxidative Stress Biomaker and Its Application to Health Maintainance. J. Clin. Biochem. Nutr..

[67] Qing H., Wong W., McGeer E.G., McGeer P.L. (2009). Lrrk2 Phosphorylates Alpha Synuclein at Serine 129: Parkinson Disease Implications. Biochem. Biophys. Res. Commun..

[68] Sotomayor-Vivas C., Hernández-Lemus E., Dorantes-Gilardi R. (2022). Linking Protein Structural and Functional Change to Mutation Using Amino Acid Networks. PLoS ONE.

[69] Rahman A.A., Morrison B.E. (2019). Contributions of VPS35 Mutations to Parkinson’s Disease. Neuroscience.

[70] Bravo-San Pedro J.M., Niso-Santano M., Gómez-Sánchez R., Pizarro-Estrella E., Aiastui-Pujana A., Gorostidi A., Climent V., López de Maturana R., Sanchez-Pernaute R., López de Munain A. (2013). The LRRK2 G2019S Mutant Exacerbates Basal Autophagy through Activation of the MEK/ERK Pathway. Cell. Mol. Life Sci..

[71] Lee C., Menozzi E., Chau K., Schapira A.H.V. (2021). Glucocerebrosidase 1 and Leucine-rich Repeat Kinase 2 in Parkinson Disease and Interplay between the Two Genes. J. Neurochem..

[72] Kluss J.H., Beilina A., Williamson C.D., Lewis P.A., Cookson M.R., Bonet-Ponce L. (2022). Lysosomal Positioning Regulates Rab10 Phosphorylation at LRRK2^+^ Lysosomes. Proc. Natl. Acad. Sci. USA.

[73] Ysselstein D., Nguyen M., Young T.J., Severino A., Schwake M., Merchant K., Krainc D. (2019). LRRK2 Kinase Activity Regulates Lysosomal Glucocerebrosidase in Neurons Derived from Parkinson’s Disease Patients. Nat. Commun..

[74] Funayama M., Nishioka K., Li Y., Hattori N. (2023). Molecular Genetics of Parkinson’s Disease: Contributions and Global Trends. J. Hum. Genet..

[75] Ortega M.A., Fraile-Martinez O., de Leon-Oliva D., Boaru D.L., Lopez-Gonzalez L., García-Montero C., Alvarez-Mon M.A., Guijarro L.G., Torres-Carranza D., Saez M.A. (2024). Autophagy in Its (Proper) Context: Molecular Basis, Biological Relevance, Pharmacological Modulation, and Lifestyle Medicine. Int. J. Biol. Sci..

[76] Cleeter M.W.J., Chau K.-Y., Gluck C., Mehta A., Hughes D.A., Duchen M., Wood N.W., Hardy J., Mark Cooper J., Schapira A.H. (2013). Glucocerebrosidase Inhibition Causes Mitochondrial Dysfunction and Free Radical Damage. Neurochem. Int..

[77] Nechushtai L., Frenkel D., Pinkas-Kramarski R. (2023). Autophagy in Parkinson’s Disease. Biomolecules.

[78] Rubilar J.C., Outeiro T.F., Klein A.D. (2024). The Lysosomal β-Glucocerebrosidase Strikes Mitochondria: Implications for Parkinson’s Therapeutics. Brain.

[79] Ge P., Dawson V.L., Dawson T.M. (2020). PINK1 and Parkin Mitochondrial Quality Control: A Source of Regional Vulnerability in Parkinson’s Disease. Mol. Neurodegener..

[80] Stone J.R., Yang S. (2006). Hydrogen Peroxide: A Signaling Messenger. Antioxid. Redox. Signal.

[81] Kamikawaji S., Ito G., Iwatsubo T. (2009). Identification of the Autophosphorylation Sites of LRRK2. Biochemistry.

[82] West A.B., Moore D.J., Choi C., Andrabi S.A., Li X., Dikeman D., Biskup S., Zhang Z., Lim K.-L., Dawson V.L. (2007). Parkinson’s Disease-Associated Mutations in LRRK2 Link Enhanced GTP-Binding and Kinase Activities to Neuronal Toxicity. Hum. Mol. Genet..

[83] Li X., Moore D.J., Xiong Y., Dawson T.M., Dawson V.L. (2010). Reevaluation of Phosphorylation Sites in the Parkinson Disease-Associated Leucine-Rich Repeat Kinase 2. J. Biol. Chem..

[84] Dzamko N., Inesta-Vaquera F., Zhang J., Xie C., Cai H., Arthur S., Tan L., Choi H., Gray N., Cohen P. (2012). The IkappaB Kinase Family Phosphorylates the Parkinson’s Disease Kinase LRRK2 at Ser935 and Ser910 during Toll-Like Receptor Signaling. PLoS ONE.

[85] Fujiwara H., Hasegawa M., Dohmae N., Kawashima A., Masliah E., Goldberg M.S., Shen J., Takio K., Iwatsubo T. (2002). α-Synuclein Is Phosphorylated in Synucleinopathy Lesions. Nat. Cell Biol..

[86] Du T., Wang L., Liu W., Zhu G., Chen Y., Zhang J. (2021). Biomarkers and the Role of α-Synuclein in Parkinson’s Disease. Front. Aging Neurosci..

[87] Mendivil-Perez M., Velez-Pardo C., Jimenez-Del-Rio M. (2016). Neuroprotective Effect of the LRRK2 Kinase Inhibitor PF-06447475 in Human Nerve-like Differentiated Cells Exposed to Oxidative Stress Stimuli: Implications for Parkinson’s Disease. Neurochem. Res..

[88] Giraldo-Berrio D., Mendivil-Perez M., Velez-Pardo C., Jimenez-Del-Rio M. (2024). Rotenone Induces a Neuropathological Phenotype in Cholinergic-like Neurons Resembling Parkinson’s Disease Dementia (PDD). Neurotox. Res..

[89] Quintero-Espinosa D.A., Sanchez-Hernandez S., Velez-Pardo C., Martin F., Jimenez-Del-Rio M. (2023). LRRK2 Knockout Confers Resistance in HEK-293 Cells to Rotenone-Induced Oxidative Stress, Mitochondrial Damage, and Apoptosis. Int. J. Mol. Sci..

[90] Hartmann A., Hunot S., Michel P.P., Muriel M.-P., Vyas S., Faucheux B.A., Mouatt-Prigent A., Turmel H., Srinivasan A., Ruberg M. (2000). Caspase-3: A Vulnerability Factor and Final Effector in Apoptotic Death of Dopaminergic Neurons in Parkinson’s Disease. Proc. Natl. Acad. Sci. USA.

[91] Monteiro L.d.B., Davanzo G.G., de Aguiar C.F., Moraes-Vieira P.M.M. (2020). Using Flow Cytometry for Mitochondrial Assays. MethodsX.

[92] Lazic S.E., Clarke-Williams C.J., Munafò M.R. (2018). What Exactly Is ‘N’ in Cell Culture and Animal Experiments?. PLoS Biol..

[93] Maayan Eshed G., Alcalay R.N. (2024). GBA1-and LRRK2-Directed Treatments: The Way Forward. Park. Relat. Disord..

[94] Auburger G., Klinkenberg M., Drost J., Marcus K., Morales-Gordo B., Kunz W.S., Brandt U., Broccoli V., Reichmann H., Gispert S. (2012). Primary Skin Fibroblasts as a Model of Parkinson’s Disease. Mol. Neurobiol..

[95] Corenblum M., McRobbie-Johnson A., Carruth E., Bernard K., Luo M., Mandarino L., Peterson S., Sans-Fuentes M., Billheimer D., Maley T. (2023). Parallel Neurodegenerative Phenotypes in Sporadic Parkinson’s Disease Fibroblasts and Midbrain Dopamine Neurons. Prog. Neurobiol..

